# Copper(II) Complexes with 1-(Isoquinolin-3-yl)heteroalkyl-2-ones: Synthesis, Structure and Evaluation of Anticancer, Antimicrobial and Antioxidant Potential

**DOI:** 10.3390/ijms25010008

**Published:** 2023-12-19

**Authors:** Łukasz Balewski, Tomasz Plech, Izabela Korona-Głowniak, Anna Hering, Małgorzata Szczesio, Andrzej Olczak, Patrick J. Bednarski, Jakub Kokoszka, Anita Kornicka

**Affiliations:** 1 Department of Chemical Technology of Drugs, Faculty of Pharmacy, Medical University of Gdansk, Gen. J. Hallera 107, 80-416 Gdańsk, Poland; lukasz.balewski@gumed.edu.pl (Ł.B.); jakub.kokoszka@gumed.edu.pl (J.K.); 2 Department of Pharmacology, Medical University of Lublin, Radziwiłłowska 11, 20-080 Lublin, Poland; tomasz.plech@umlub.pl; 3 Department of Pharmaceutical Microbiology, Medical University of Lublin, Chodźki 1, 20-093 Lublin, Poland; iza.glowniak@umlub.pl; 4 Department of Biology and Pharmaceutical Botany, Faculty of Pharmacy, Medical University of Gdansk, Gen. J. Hallera 107, 80-416 Gdańsk, Poland; anna.hering@gumed.edu.pl; 5 Institute of General and Ecological Chemistry, Faculty of Chemistry, Lodz University of Technology, Żeromskiego 116, 90-924 Łódź, Poland; malgorzata.szczesio@p.lodz.pl (M.S.); andrzej.olczak@p.lodz.pl (A.O.); 6 Department of Pharmaceutical and Medicinal Chemistry, Institute of Pharmacy, University of Greifswald, F.-L. Jahn Strasse 17, D-17489 Greifswald, Germany; bednarsk@uni-greifswald.de

**Keywords:** isoquinoline derivatives, copper(II) complexes, synthesis, structural, stability studies, antitumor, antimicrobial, antioxidant properties, ADME analysis

## Abstract

Four copper(II) complexes, **C1**–**4**, derived from 1-(isoquinolin-3-yl)heteroalkyl-2-one ligands **L1**–**4** were synthesized and characterized using an elemental analysis, IR spectroscopic data as well as single crystal X-ray diffraction data for complex **C1**. The stability of complexes **C1**–**4** under conditions mimicking the physiological environment was estimated using UV-Vis spectrophotometry. The antiproliferative activity of both ligands **L1**–**4** and copper(II) compounds **C1**–**4** were evaluated using an MTT assay on four human cancer cell lines, A375 (melanoma), HepG2 (hepatoma), LS-180 (colon cancer) and T98G (glioblastoma), and a non-cancerous cell line, CCD-1059Sk (human normal skin fibroblasts). Complexes **C1**–**4** showed greater potency against HepG2, LS180 and T98G cancer cell lines than *etoposide* (IC_50_ = 5.04–14.89 μg/mL vs. IC_50_ = 43.21–>100 μg/mL), while free ligands **L1**–**4** remained inactive in all cell lines. The prominent copper(II) compound **C2** appeared to be more selective towards cancer cells compared with normal cells than compounds **C1**, **C3** and **C4**. The treatment of HepG2 and T98G cells with complex **C2** resulted in sub-G1 and G2/M cell cycle arrest, respectively, which was accompanied by DNA degradation. Moreover, the non-cytotoxic doses of **C2** synergistically enhanced the cytotoxic effects of chemotherapeutic drugs, including *etoposide*, *5-fluorouracil* and *temozolomide*, in HepG2 and T98G cells. The antimicrobial activities of ligands **L2**–**4** and their copper(II) complexes **C2**–**4** were evaluated using different types of Gram-positive bacteria, Gram-negative bacteria and yeast species. No correlation was found between the results of the antiproliferative and antimicrobial experiments. The antioxidant activities of all compounds were determined using the DPPH and ABTS radical scavenging methods. Antiradical tests revealed that among the investigated compounds, copper(II) complex **C4** possessed the strongest antioxidant properties. Finally, the ADME technique was used to determine the physicochemical and drug-likeness properties of the obtained complexes.

## 1. Introduction

Since metal-based compounds play remarkable roles as therapeutic and diagnostic agents, the search for novel metallopharmaceuticals represents an area of significant interest in the field of medicinal chemistry [1,2]. The therapeutic potential of metal complexes has a long history; however, the discovery of the bacteriostatic and anticancer activity ruthenium(II) phenanthroline complexes by Francis Dwyer, followed by the discovery of the anticancer properties of *cis*-diaminodichloridoplatinum(II)—*cisplatin* by Barnet Rosenberg, was a milestone in the development of metal-containing drugs [1]. Among clinically approved anticancer chemotherapeutics, platinum agents such as *cisplatin* and its derivatives, e.g., *carboplatin oxaliplatin* and *picoplatin*, are still the most prominent drugs used in the treatment of solid cancers [3]. Nonetheless, despite the evident success of *cisplatin* and its analogues in medicine, their progress in clinical application is limited due to their well-known drawbacks, such as low solubility, severe side effects, including nephrotoxicity and neurotoxicity, and the intrinsic or acquired resistance of cancer cells to platinum-containing drugs [4,5,6,7]. Consequently, with the emergence of many biomedical applications of other transition metal complexes, including anticancer and antimicrobial agents, attention is shifting beyond platinum-based compounds [1,8,9,10,11]. The preclinical studies provide evidence that non-platinum agents have the potential to circumvent the problem of chemoresistance and the toxicity of platinum-based agents by exhibiting different specific modes of action, reduced undesirable effects and the ability to overcome drug-resistance mechanisms [10,12,13]. 

Antitumor agents based on endogenous metals such as cobalt, zinc, iron or copper were found to be less toxic compared to platinum analogues [14]. Among them, copper(II)-containing coordination compounds have attracted considerable interest due to the significant role of copper in cancer [15,16]. The copper ion is involved in essential processes of cancer progression such as cell proliferation, angiogenesis or metastasis, and it provides various mechanisms of antitumor action [17,18,19]. For example, the antiproliferative effect of copper(II) complexes results from the inhibition of the activities of enzymes, which play a pivotal role in cancer therapy, e.g., protein disulfide isomerase (PDI) [20], topoisomerases I and II [21,22,23], telomerase [24] or proteasome [25,26,27], as well as DNA intercalation [28,29,30] and DNA degradation [31,32]. Moreover, the antitumor activity of copper compounds may also be the consequence of their ability to induce apoptosis [33,34] and non-apoptotic cell death—paraptosis [25,35]—reactive oxygen species (ROS) formation that triggers tumor cell death [36,37], and antiangiogenic properties [38]. In turn, the copper(II) complex with *disulfiram*, a drug used in humans to treat alcoholism with great potential for the treatment of human cancers [39], was found to be capable of reversing the drug resistance of *doxorubicin* (ADM)-resistant acute leukemia cell lines by the induction of apoptosis [40]. Additionally, it is well known that the copper coordination in organic compounds may lead to higher antitumor activity [23,41], selectivity [23] and reduced toxicity [42] compared to the free ligands. In this line, it is worth noting that the uptake of copper(II) complexes by cancer cells is higher than that by normal cells [38,43]. In addition, copper(II) complexes may exhibit a different response to cancer cells than to non-cancerous cells [44]. Overall, copper(II)-containing coordination compounds have emerged as a promising class of therapeutic anticancer agents with various mechanisms of action and the potential to overcome drug resistance [17,20,45]. 

It should be noted that the therapeutic potency of copper(II) complexes is not limited to anticancer activity. These compounds have also gained much interest due to their anti-inflammatory and antioxidant properties [46,47,48], antiviral properties [49,50] or antibiofilm and antimicrobial [51,52] activities. The latter is a multi-faceted process, although the main mechanism of the bactericidal effect is the formation of ROS, causing irreversible damage to membranes [51].

Isoquinoline-containing compounds are of scientific interest due to their fluorescent properties and broad spectrum of biological activities including antihypertensive [53], anti-inflammatory and analgesic [54] or antioxidant [55] activities, as well as their ability to act as antidepressant and antipsychotic [56] agents. Moreover, the importance of isoquinoline scaffold in drug design [57,58] has also led to the development of bioactive compounds with antimalarial [59], antifungal and antibacterial effects [60,61]. In addition, isoquinoline derivatives constitute an important source of novel anticancer agents that may exert their biological activities through various mechanisms such as apoptosis, DNA fragmentation, the inhibition of tubulin polymerization, induced cell cycle arrest and the interruption of cell migration [62,63]. On the other hand, the research on the biological activities of their metal complexes, especially copper(II)-coordination compounds, is not very extensive.

**Figure 1:**
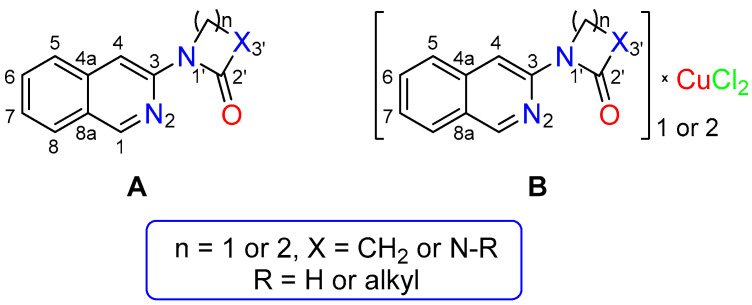
1-(isoquinolin-3-yl)heteroalkyl-2-one ligands (**A**) and copper(II) complexes of 1-(isoquinolin-3-yl)-heteroalkyl-2-ones (**B**).

Our previous work indicated that the newly synthesized 1-(isoquinolin-3-yl)heteroalkyl-2-ones of type **A** (Figure 1) possess promising fluorescent properties [64]. In the present work, as a continuation of a research program on the chemistry and biological activity of copper(II) complexes undertaken in our laboratory [64,65,66,67,68], we wish to report the results of studies on the reactions of the above-mentioned isoquinoline derivatives **A** with copper(II) chloride, an X-ray structure determination of the complexes obtained of type **B** (Figure 1), as well as the results of the evaluation of their anticancer, antimicrobial and antioxidant potential. Furthermore, to confirm the importance of copper coordination in organic ligands, the biological properties of the free ligands **A** were also evaluated.

## 2. Results and Discussion

### 2.1. Chemistry

#### 2.1.1. Synthesis of 1-(Isoquinolin-3-yl)heteroalkyl-2-one Ligands **L1**–**4**

**Scheme 1:**
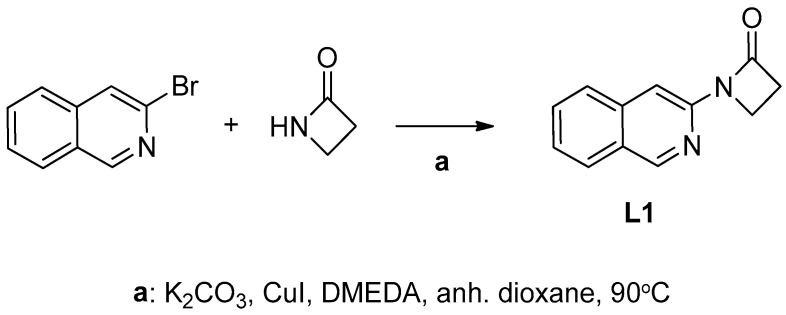
Synthesis of 1-(isoquinolin-3-yl)azetidin-2-one ligand (**L1**).

Ligand 1-(isoquinolin-3-yl)azetidin-2-one (**L1**) was synthesized according to a previously described procedure involving copper-catalyzed Goldberg–Ullmann-type coupling of 3-bromoisoquinoline with azetidin-2-one [64]. These reactions were carried out in anhydrous dioxane or *n*-butyl alcohol in the presence of a base, copper(I) iodide and *N*,*N*-dimethylethylenediamine (Scheme 1). 

In turn, the use of isoquinoline *N*-oxide and 2-chloroimidazoline as substrates allowed us to obtain the ligand-containing cyclic urea moiety—1-(isoquinolin-3-yl)imidazolidin-2-one (**L2**)—as a major product [69,70]. 

**Scheme 2:**
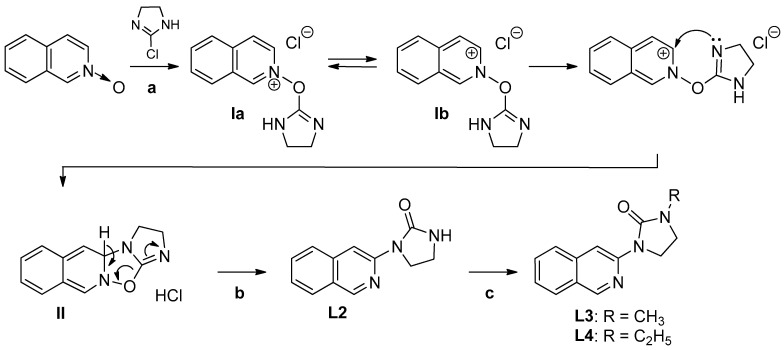
Synthesis of ligand **L2** in the reaction of isoquinoline *N*-oxide with 2-chloroimidazoline and *N*-alkylated analogous **L3** and **L4**.

The reaction leading to compound **L2** proceeds exothermically and spontaneously in a polar aprotic solvent (dichloromethane or chloroform) at an ambient temperature, and it involves a few steps (Scheme 2). Firstly, isoquinoline *N*-oxide attacks 2-chloroimidazoline to form 2-((4,5-dihydro-1*H*-imidazol-2-yl)oxy)isoquinolin-2-ium chloride (**Ia**), which, in the mesomeric form **Ib**, possesses a nucleophilic carbon atom in position 3 that is susceptible to the intramolecular attack of the nitrogen atom of the imidazoline ring. In the next step, the simultaneous re-aromatization of intermediate—3,4*a*-dihydro-2*H*-imidazo[1′,2′:4,5][1,2,4]oxadiazolo[2,3-*b*]isoquinoline (**II**) followed by the elimination of hydrogen chloride leads to the formation of the desired 1-(isoquinolin-3-yl)imidazolidin-2-one (Scheme 2). Upon the treatment of compound **L2** with methyl or ethyl iodide in the presence of sodium hydroxide, the corresponding *N*-alkylated ligands **L3** and **L4** were synthesized in acceptable yields (Scheme 2).

#### 2.1.2. Synthesis of Copper(II) Complexes of 1-(Isoquinolin-3-yl)heteroalkyl-2-ones **C1**–**4**

Copper(II) complexes **C1**–**4** were prepared through the reaction of copper(II) chloride dihydrate with previously described 1-(isoquinolin-3-yl)heteroalkyl-2-ones **L1**–**4** [64]. For the preparation of metal complexes that are stable under physiological conditions, reactions were carried out in dimethylformamide (DMF) containing 0.5% water and the dihydrate of copper(II) salt. The use of dimethylformamide as a solvent had a positive effect on the purity and further isolation of the desired copper(II) complexes. The formation of precipitate or crystals of green or brown copper(II) complexes was observed at room temperature over a period of 3 to 12 days. After the required time, the products—copper(II) complexes—were separated by filtration. 

Firstly, we began by studying the equimolar ratio of ligands **L1**–**4** and copper(II) salt in 99.5% dimethylformamide. It was found that for the copper(II) complexes **C3** and **C4**, a stoichiometric ratio of the ligands **L3** and **L4** in terms of yields was the most important factor. In the case of copper(II) complexes **C1** and **C2**, two molecules of a neutral bidentate ligand **L1** or **L2** can coordinate with the copper(II) ion. Thus, a 2-fold excess of ligands **L1** or **L2** resulted in the creation of tetra-coordinate mononuclear copper(II) complexes **C1** and **C2**, respectively. 

**Scheme 3:**
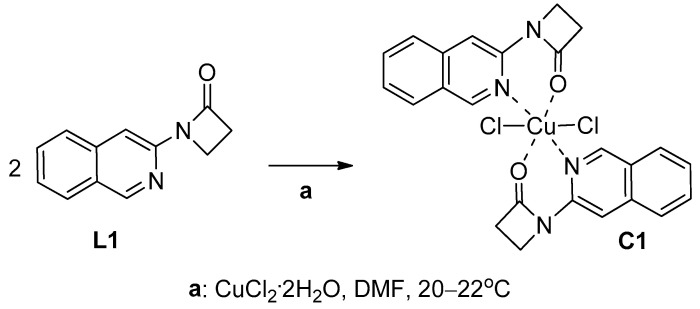
Synthesis of copper(II) complex **C1** in the reaction of ligand **L1** with copper(II) chloride dihydrate in dimethylformamide.

Mononuclear complex **C1** (L_2_CuCl_2_) was obtained in the reaction of ligand **L1** with copper(II) chloride dihydrate in a 1:1 molar ratio through the slow evaporation of the solvent at room temperature (Scheme 3). 

**Scheme 4:**
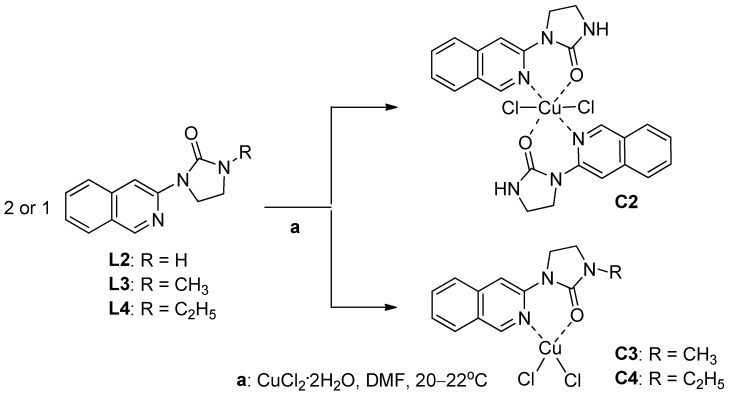
Synthesis of copper(II) complexes **C2**, **C3** and **C4** in the reaction of **L2**, **L3** and **L4** with copper(II) chloride dihydrate in dimethylformamide.

The coordination compounds **C2**, **C3** and **C4** were obtained in acceptable yields with analogous reaction conditions and copper(II) salt stoichiometries (Scheme 4). It should be emphasized that the ligand unsubstituted at the nitrogen atom in position 3 of the imidazolidin-2-one; derivative **L2** formed a neutral mononuclear chelate **C2** (L_2_CuCl_2_), while the *N*^3^-substituted ligands **L3** and **L4** allowed the preparation of brown-green bidentate *N*,*O*-chelates **C3** and **C4** with the structure LCuCl_2_.

Summing up, the efficiency of the complexation reactions, calculated as the ratio of the achieved yield to the theoretical yield, was approximately twice higher (61–68%) in the case of the bidentate *N*,*O*-chelates **C3** and **C4** (LCuCl_2_) than the mononuclear complexes **C1** and **C2** with the L_2_CuCl_2_ structure (27–33%).

### 2.2. Structural Analysis of Copper(II) Complexes ***C1**–**4***

The structures of copper(II) complexes **C1**–**4** were confirmed using an elemental analysis and infrared spectroscopic data. Moreover, the crystal structure of copper(II) complex **C1** was determined using X-ray crystallography. It should be mentioned that the presence of an unpaired electron attributed to the copper(II) ion in complexes **C1**–**4** results in their paramagnetic properties; thus, the nuclear magnetic resonance (NMR) spectra of compounds **C1**–**4** cannot be recorded. 

#### 2.2.1. Infrared Spectra

In the infrared spectra of copper(II) complexes **C1**, **C2**, **C3** and **C4** stretching vibrations of the carbonyl group (C=O) were observed in the range of 1639 to 1751 cm^−1^ . It should be noted that the IR spectra of the copper(II) complexes showed the characteristic shifts of functional group absorptions, confirming their involvement in the chelation of a metal ion. Hence, in the case of synthesized complexes, shifted stretching bands were observed for the C=O group of the lactam (**C1**) or cyclic urea ring (**C2**, **C3** and **C4**), and the C=N moiety of the isoquinoline ring (Figures S1–S8, Supplementary Materials). 

**Figure 2:**
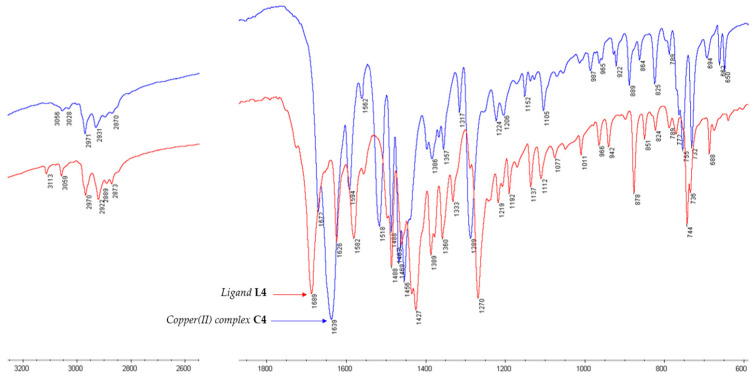
FTIR spectra of ligand **L4** (red line) and corresponding copper(II) complex (LCuCl_2_) **C4** (blue line).

For example, the IR spectrum registered for dichloro[1-ethyl-3-(isoquinolin-3-yl)imidazolidin-2-one]copper(II) (**C4**)—superimposed on the IR spectrum of the parent ligand **L4**—indicated a shift in the carbonyl stretching band by a value of 50 cm^−1^ towards lower values of wavelengths (1689 cm^−1^ → 1639 cm^−1^) (Figure 2).

#### 2.2.2. X-ray Crystallographic Studies

The crystal data, data collection and structure refinement details are summarized in Table 1. 

**Figure 3:**
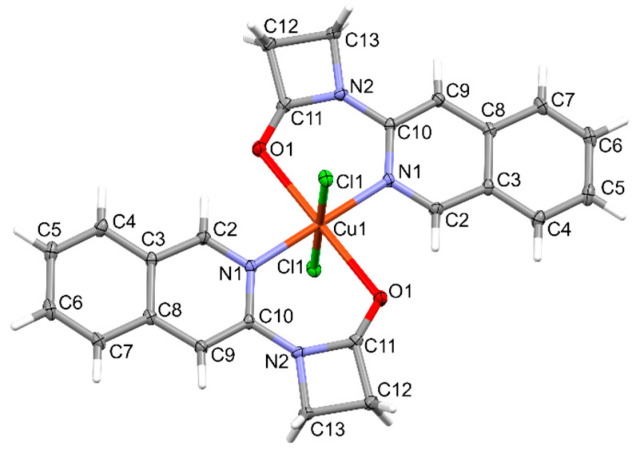
Structural representation and atom-numbering scheme. Thermal ellipsoids are drawn at the 50% probability level.

In the crystal structure of complex **C1**, the organic ligand **L1** is coordinated in the bidentate manner via the oxygen atom of the carbonyl group and the nitrogen atom of the isoquinoline ring, forming a six-membered chelate cycle (Figure 3). 

**Figure 4:**
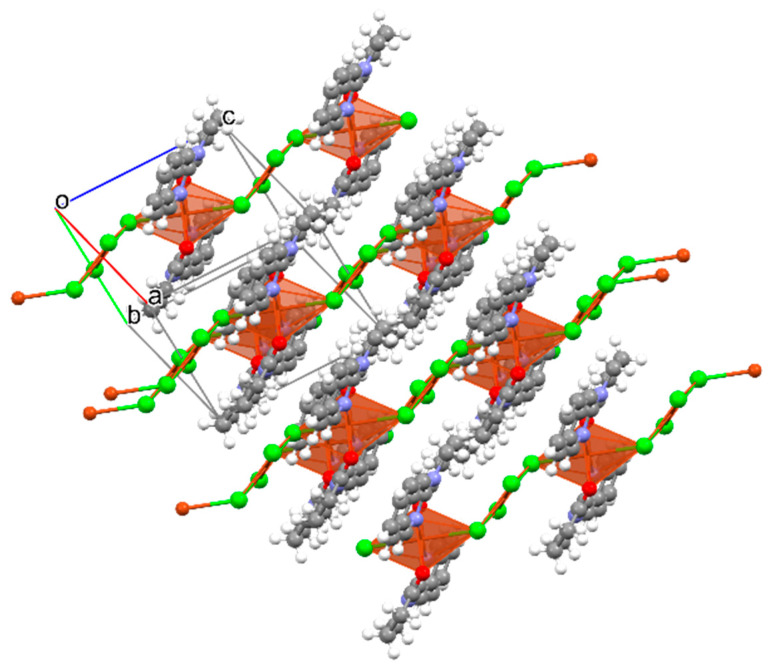
Crystal packing of copper(II) complex **C1** in the unit cell.

Coordinated ligands are in *trans* position to each other. The copper ion is in a distorted octahedral environment with two N atoms and two O atoms from two ligands in the equational plane and two Cl donors in the opposite axial sites (Figure 4). The copper ions reside in the center of the octahedron, in which the bond lengths are Cu1 − N1 = 2.0666 (12)Å, Cu1 − O1 = 2.4210 (11) Å and Cu1 − Cl1 = 2.3051 (4) Å.

There are no strong hydrogen bonds in the **C1** structure. There are hydrogen bonds of the C-H…Cl(O) type. The C-H…O bond stabilizes the coordination fragment (Table 2). However, the C-H…Cl hydrogen bond stabilizes the packing. 

Some additional geometrical details can be found in the Supplementary Materials (Tables S1–S3).

#### 2.2.3. Molecular Modeling Studies of Ligands **L1** and **L2**

**Figure 5:**
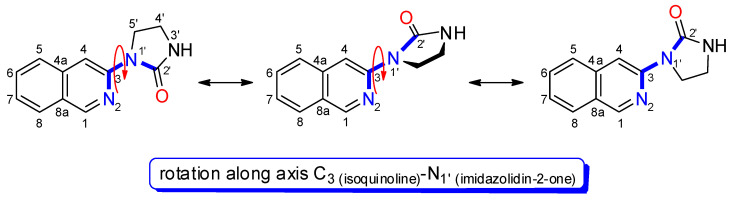
Structures of possible rotamers of ligand **L2** obtained by rotation along the C_3_-N_1′_ axis.

Our previous X-ray studies indicated that in the crystal state, the compound **L2** adopts the *E* conformation, which is probably stabilized by intramolecular C-H⋯O (2.26 Å). The molecules of ligand **L2** are connected by pairs of N-H⋯O hydrogen bonds (1.99 Å) between two imidazolidin-2-one ring fragments with the formation of centrosymmetric dimers [64]. The many possible rotamers of the representative ligand—1-(isoquinolin-3-yl)imidazolidin-2-one (**L2**)—can be generated from the rotation around the bond axis C3_(isoquinoline)_-N1′_(imidazolin-2-one)_ (Figure 5). 

In this study, the X-ray diffraction analysis results obtained for copper(II) complex **C1** showed that two molecules of ligand **L1** exist in the conformation *E*. For this reason, we decided to perform quantum chemical calculations to gain a better understanding of the structure of the ligands **L1**–**4**. We assumed that the formation of the copper(II) complexes of ligands **L1**–**4** requires a rotation of their conformation from *E* to *Z*. 

The structures of the selected two ligands **L1** and **L2** were optimized in a polar solvent (DMF) by using the Spartan program suite (Spartan version 14 V 1.1.4.). The possible conformers of compounds **L1** and **L2** were calculated at the B3LYP/6.31G** level of theory [71,72,73]. It should be mentioned that B3LYP—the so-called ‘hybrid functional’—is one of the most popular DFT functionals used for the prediction of the physicochemical properties of molecules in in silico drug design [74].

**Figure 6:**
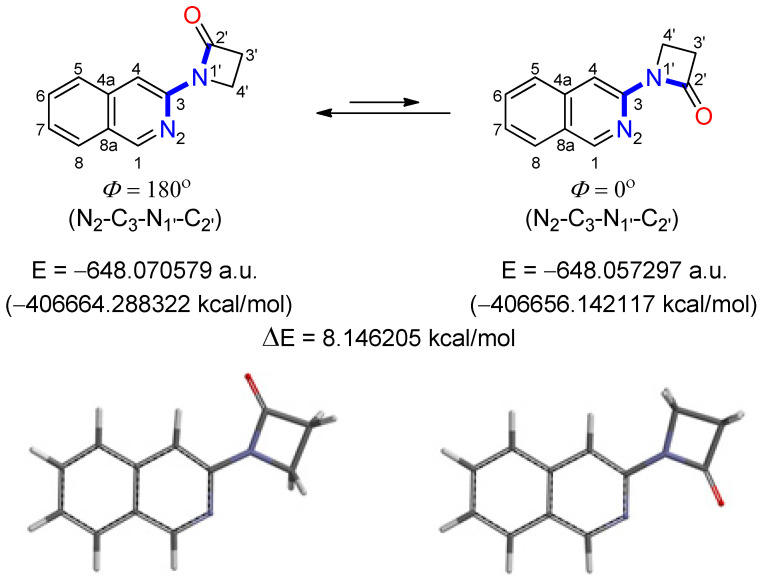
Structures of two possible rotamers of ligand **L1** and corresponding electronic energies (E, a.u.) and relative energy (ΔE, kcal/mol) calculated in DMF at B3LYP/6.31G** level of theory.

In the case of ligand **L1**, the structure with a torsion angle (N_2_-C_3_-N_1′_-C_2′_, *Φ* = 180°) was proven to be the lowest energy rotameric form in the DMF solution (*E* conformation of molecule) (Figure 6). The energy difference between the *E* conformation and its rotamer in the *Z* conformation (N_2_-C_3_-N_1′_-C_2′_, *Φ* = 0) was calculated to be ΔE = 8.146 kcal/mol. Based on this, it may be concluded that the barrier to the C_3_-N_1′_ bond rotation is low, and it is easy to overcome the energy barrier under normal conditions. This suggests that rotamers having different torsion angles may exist together in the solution at room temperature. In the *Z* conformation of 1-(isoquinolin-3-yl)azetidin-2-one (**L1**), the position of the nitrogen and oxygen atoms of the two heterocyclic rings is favored for the chelation of copper(II) ions. 

**Figure 7:**
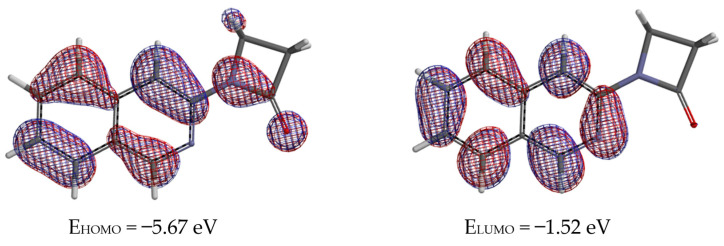
Orbital diagrams of HOMO (**left**) and LUMO (**right**) for optimized structure of conformer ligand L1 with torsion angle *Φ* = 0°.

In the case of ligand **L1** in its *E* conformation with torsion angle *Φ* = 0, the highest occupied molecular orbital (E_HOMO_ = −5.67 eV) is confined to carbon atoms C_3_–C_8_ of the isoquinoline ring, and nitrogen (N_1′_) and carbon (C_2′_) atoms of the azetydin-2-one system, while the frontier orbital LUMO (E_LUMO_ = −1.52 eV) is located mostly on the entire isoquinoline scaffold (Figure 7). The calculated HOMO-LUMO energy gap for ligand **L1** in conformation with the torsion angle *Φ* = 0 (E_g_ = 4.15 eV) is lower than the energy gap obtained for its *Φ* = 180 rotamer (E_g_ = 4.27 eV). This may suggest the higher reactivity of conformer **L1** with the torsion angle *Φ* = 0 towards bonding with transient metals such as copper. 

**Figure 8:**
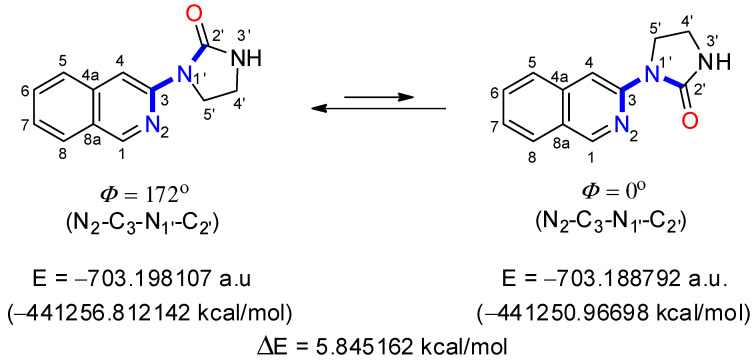
Structures of two possible rotamers of ligand **L2**, and corresponding electronic energies (E, a.u.) and relative energy (ΔE, kcal/mol) calculated in DMF at B3LYP/6.31G** level of theory.

Similarly, the in silico data for ligand **L2** revealed that conformer with torsion angle N_2_-C_3_-N_1′_-C_2′_ at about *Φ* = 172 was calculated to be slightly lower in energy (E = −441,256.812142 kcal/mol) than the conformer with torsion angle *Φ* = 0 (E = −441,250.96698 kcal/mol) (Figure 8). The energy difference between these two rotamers was estimated to be ΔE = 5.845 kcal/mol. Moreover, based on calculated dipole moments, the conformer with torsion angle *Φ* = 0 (μ = 6.60 debye)—favored for the binding of copper(II) ions—would be predicted to prevail over the second one (*Φ* = 172, μ = 3.23 debye) in a polar solvent such as dimethylformamide. 

Based on this, it may be concluded that for ligands **L1** and **L2**, the barrier to the C_3_-N_1′_ bond rotation is low, and it is easy to overcome the energy barrier under normal conditions (at room temperature) and in polar solvents (dimethylformamide). This suggests that rotamers of ligands **L1**–**4** having different torsion angles may exist in the solution at room temperature. In their *Z* conformations, the position of the atoms having donating properties is favored for the binding of copper(II) ions. Therefore, forming a six-membered chelate ring requires energy, which can be compensated for by creating novel bonds involving copper and nitrogen or oxygen atoms: Cu^2+^---N=C and Cu^2+^---O=C. 

### 2.3. Stability Studies of Copper(II) Complexes ***C1**–**4*** in Aqueous Buffer

The stability testing of the synthesized compounds must validate the biological results by ensuring that the compound remains biologically active over time. It should be emphasized that the limited stability of drug candidates in physiological pH ranges prevents their use in vivo. In the case of metal complexes, this is a crucial aspect due to the fact that they may dissociate under physiological conditions, releasing the metal ions and the free ligands.

To address this question and confirm the validity of the biological results, we performed UV-vis stability measurements of copper(II) complexes **C1**–**4** under conditions that mimic the physiological environment (phosphate buffered aqueous solution, pH = 7.4, 37 °C). An increase in absorbance during the measurements may indicate the release of free ligands, whereas a decrease in absorbance shows the precipitation of the complex from the buffer solution. 

Firstly, copper(II) complexes were dissolved in 99.5% DMF at a concentration of 4 mM. These solutions were diluted into a 100 mM potassium phosphate buffer with pH 7.4 to a final concentration of 40 μM of copper(II) complex in a quartz cuvette at a temperature of 37 °C. To identify very small changes in the UV-vis spectra, difference spectra between λ = 250–600 nm were recorded every 10 min with a diode array photometer over the course of 3 h at 37 °C. The difference spectra were obtained by subtracting the spectrum at time = 0 from each of the following recorded spectra between λ = 250 and 600 nm. 

The tested copper(II) complexes **C1**–**4** did not show noticeable changes in their time-dependent difference spectra over 3 h of incubation in the phosphate-buffered aqueous solution (pH 7.4, 37 °C). It was observed that the intensity did not change during the experiments. It is also worth noting the lack of isosbestic points in the range of 250 and 600 nm. The presence of isosbestic points indicates a possibility of reaction in the buffered solution, for example, ligand exchange. Thus, the complexes **C1**–**4** appear stable under biologically similar conditions with no apparent loss of the Cu(II) from the ligand. 

**Figure 9:**
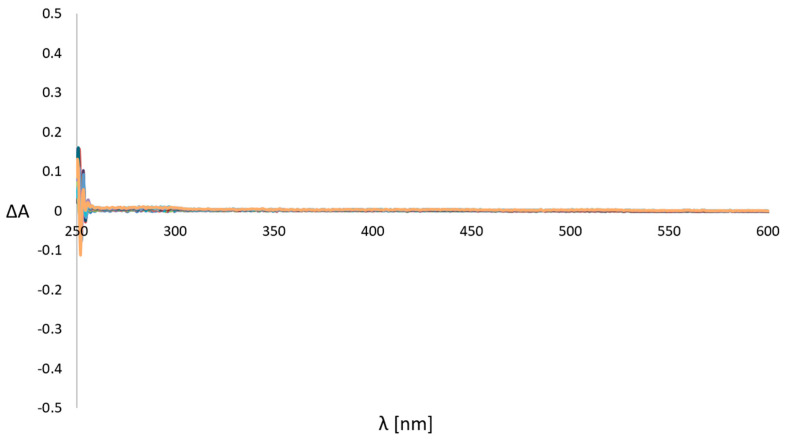
Time-dependent UV-vis difference spectra of dichloro{*bis*[1-(isoquinolin-3-yl)azetidin-2-one]}copper(II) (**C1**) over 3 h at pH = 7.4 and 37 °C.

Figure 9 presents the time-dependent UV-Vis spectra of the representative copper(II) complex **C1**. The UV-vis difference spectra of copper(II) complexes **C2**, **C3** and **C4** are shown in the supporting information (Figures S9–S12, Supplementary Materials).

### 2.4. Biological Evaluation

#### 2.4.1. In Vitro Cytotoxic Activity

The in vitro cytotoxic activities of free ligands **L1**–**4** and the corresponding copper(II) complexes **C1**–**4** were evaluated against four human cancer cell lines, namely melanoma A375, hepatoma HepG2, colon cancer LS-180 and glioblastoma T98G. To establish the selectivity towards cancer cell lines, the investigated compounds were also tested on a non-cancerous human skin fibroblast cell line—CCD-1059-Sk. 

From the results presented in Table 3, it is apparent that free ligands **L1**, **L2**, **L3** and **L4** were inactive in all cell lines up to 200 μg/mL, while their copper(II) complexes **C1**, **C2**, **C3** and **C4** exhibited remarkable growth inhibitory potency towards cancer cell lines (IC_50_ values ranging from 5.04 μg/mL to 37.97 μg/mL) compared with the widely used anticancer agent—*etoposide* (IC_50_ values between 10.20 and >100 μg/mL). 

It should be noted that copper(II) compounds **C1**–**4** showed several times greater effectiveness against cancer cell lines than the reference drug (IC_50_ = 5.04–14.89 μg/mL vs. IC_50_ = 43.21–>100 μg/mL); the exception to this was the melanoma A375 cell line, which was the least susceptible to the effect of the tested complexes (IC_50_ = 22.78–37.97 μg/mL vs. IC_50_ = 10.20 μg/mL). Among the tested complexes, compound **C2** bearing imidazolidin-2-one moiety was found to be the most potent on the HepG2 and T98G cancer cell lines (Table 3). Its analogues bearing the methyl or ethyl substituent at the R position of the imidazolidin-2-one scaffold (**C3**: R = CH_3_; **C4**: R = C_2_H_5_) displayed slightly weaker antiproliferative activities (IC_50_ = 5.04–6.97 μg/mL vs. IC_50_ = 6.72–12.00 μg/mL). Furthermore, complex **C1** with an azetidin-2-one functionality showed a comparable level of ability to inhibit the growth of the HepG2 and T98G cancer cell lines with the complexes **C3** and **C4** featuring the imidazolidin-2-one moiety (IC_50_ = 8.25–14.89 μg/mL vs. IC_50_ = 6.72–12.00 μg/mL), although compound **C1** turned out to be the least active in all cancer cell lines (IC_50_ = 14.89–37.97 μg/mL) (Table 3).

Summing up, the data presented here confirmed the hypothesis that the introduction of a metal ion into organic ligands may have a beneficial effect on the anticancer potential [11,23,38,43,75]. 

As evidenced in Table 3, the cytotoxic potency of the investigated copper(II) complexes **C1**, **C2**, **C3** and **C4** was also observed in a non-cancerous cell line, CCD-1059-Sk (IC_50_ values ranging from 18.25 μg/mL to 25.17 μg/mL). Nevertheless, when individual cell lines such as HepG2, LS180 and T98G were compared with CCD-1059-Sk, a moderate degree of selectivity for compounds **C2**, **C3** and **C4** became apparent (IC_50_ = 5.04–12.00 μg/mL vs. IC_50_ = 15.03–25.17 μg/mL). In this regard, the most active Cu(II) complex **C2** was characterized by the greatest selective effect on the HepG2, LS180 and T98G cancer cell lines, with the selectivity index (SI) ranging from 3.14 (T98G) to 4.33 (HepG2). 

#### 2.4.2. Cell Cycle Analysis

**Figure 10:**
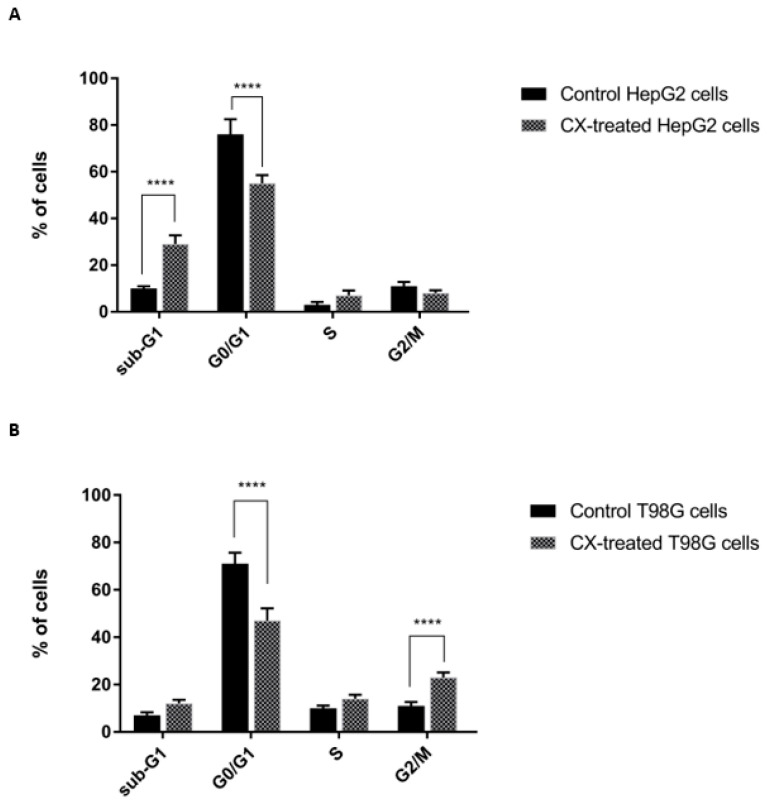
Cell cycle analysis of HepG2 (**A**) and T98G cells (**B**) incubated for 24 h with copper(II) complex **C2** (**CX**) at its IC_50_ concentration. Results are expressed as means ± SEM. Statistical significance was designated as **** when *p* < 0.0001 (vs. control cells) using ANOVA analysis followed by Tukey’s post hoc test.

Since copper(II) complex **C2** most effectively inhibited the growth of HepG2 and T98G cancer cells, its effect on cell cycle progression was examined by using the cytometry method (Figure 10). Interestingly, this compound revealed a distinct effect on the cell cycle progression of HepG2 and T98G cells. The growth inhibition of HepG2 cells was associated with cell cycle arrest in the sub-G1 phase, showing low-molecular-weight fragments of DNA as the evidence of apoptosis (Figure 10A). When analyzing the DNA content in the sub-G1 phase, a significant increase (*p* < 0.0001) from 10% in the control cells to 29% in the **C2**(**CX**)-treated HepG2 cells was observed. The identification of the occurrence of apoptosis on the basis of the elevated number of cells in the sub-G1 phase relies on the principle that degraded DNA fragments (i.e., early signs of apoptosis) are released from cells, which results in the increased number of cells possessing a reduced DNA content. Consecutively, the cytotoxic effect of **C2** (**CX**) on the T98G cells resulted from cell cycle arrest in the G2/M phase, which indicates considerable DNA damages that are unable to be repaired before mitosis (Figure 10B). 

#### 2.4.3. Interaction of Copper(II) Complex **C2** with Clinically Used Anticancer Agents 

**Figure 11:**
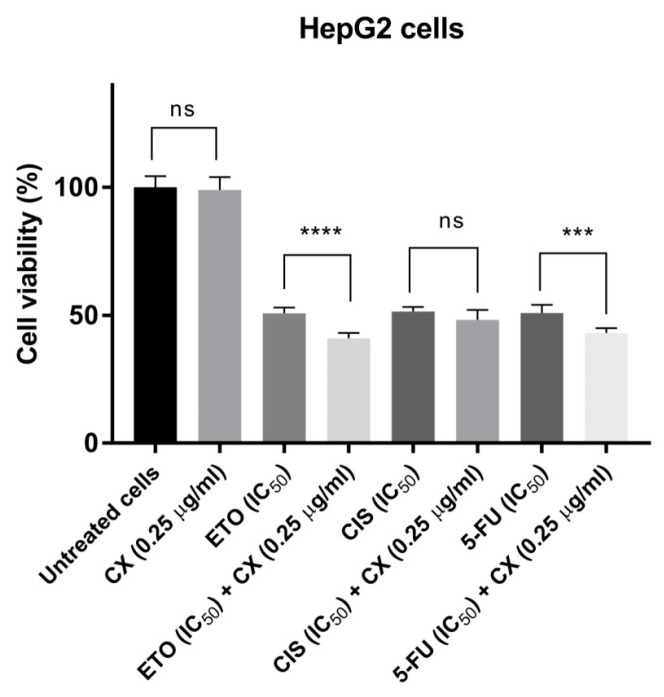
Interactions between copper(II) complex **C2** (**CX**) and anticancer drugs, *etoposide* (**ETO**), *cisplatin* (**CIS**) and *5-fluorouracil* (**5-FU**), examined on HepG2 cells using MTT assay. **CX** was tested at the highest concentration that did not affect the viability of HepG2 cells (i.e., 0.25 µg/mL). Chemotherapeutics (**ETO**, **CIS**, **5-FU**) were tested at their IC_50_ concentrations. Statistical analysis: one-way ANOVA with Tukey’s post hoc test; ns—not significant; *** *p* < 0.001; **** *p* < 0.0001.

**Figure 12:**
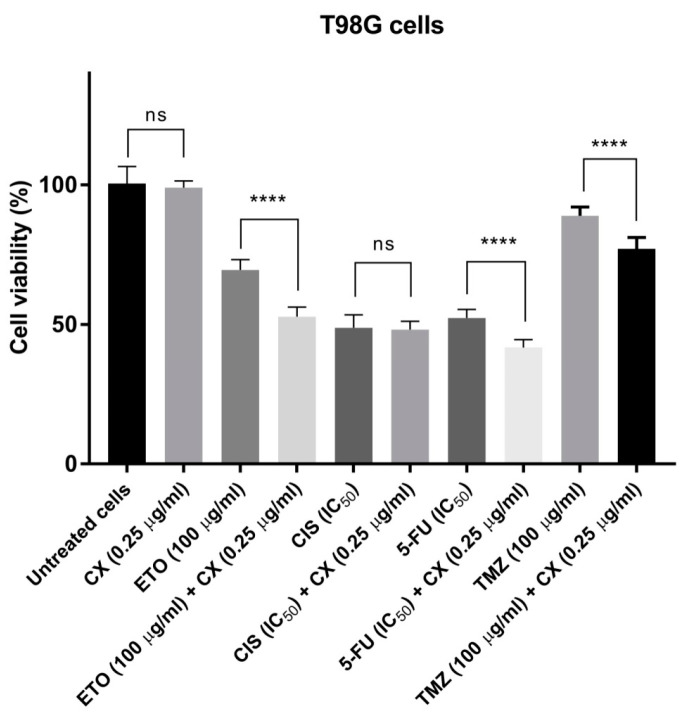
Interactions between copper(II) complex **C2** (**CX**) and anticancer drugs, *etoposide* (**ETO**), *cisplatin* (**CIS**) and *5-fluorouracil* (**5-FU**), examined on T98G cells using MTT assay. **CX** was tested at the highest concentration that did not affect the viability of T98G cells (i.e., 0.25 µg/mL). Chemotherapeutics (**ETO**, **CIS**, **5-FU**) were tested at their IC_50_ concentrations. Statistical analysis: one-way ANOVA with Tukey’s post hoc test; ns—not significant; **** *p* < 0.0001.

Possible interactions between anticancer drugs should be an important consideration among patients undergoing antineoplastic therapy since they are exposed to several types of treatments, each including a number of drugs. As most of them have a narrow therapeutic index, it is important to examine the possible new strategies that can increase the clinical outcomes by using lower doses of the currently available anticancer drugs. Such co-treatments can also be an effective strategy for overcoming resistance in cancer therapy. In our studies, copper(II) complex **C2**, which exhibited the most potent effect against HepG2 and T98G cancer cells, was selected to examine the possible synergism with clinically used anticancer agents. The combinations of **C2** (**CX**) and *etoposide* (**ETO**), *cisplatin* (**CIS**) and *5-fluorouracil* (**5-FU**) were evaluated against both of the cell lines (Figure 11 and Figure 12). Additionally, the combination of **C2** (**CX**) and *temozolomide* (**TMZ**)—a chemotherapeutic agent being used as a first-line treatment for glioblastoma—was tested on the T98G glioblastoma cell line (Figure 11). 

The investigated drugs represent different mechanisms of anticancer activity, including human DNA topoisomerase IIα inhibitors (**ETO**) [76], alkylating agents (**CIS**, **TMZ**) [77,78], thymidylate synthase inhibitors and antimetabolites (**5-FU**) [79]. Compound **C2** (**CX)**, at the concentration that did not inhibit the growth of cancer cells (i.e., 0.25 µg/mL), statistically significantly improved the cytotoxic effects of **ETO**, **5-FU** and **TMZ** against HepG2 and T98G cells (Figure 11 and Figure 12). 

To better understand the mechanism of synergism between **C2** and the clinically used drugs, it is important to gain insight into the mechanism of action of **C2** alone. Usually, beneficial drug–drug interactions (synergism) can be expected when the components of the drug mixture act via different mechanisms. 

In this context, it should be mentioned that metal complexes with isoquinoline derivatives may exert anticancer properties through S-phase cell cycle arrest by the up-regulation of p53, p27 and p21 proteins and the down-regulation of cyclin A and cyclin E [80], mitochondrial (intrinsic) pathway-dependent apoptosis [81,82], caspase-3 activation triggering apoptosis [83], the inhibition of telomerase activity [75] as well as DNA intercalation [81,83]. 

In our studies, the cytotoxic activity of copper(II) complexes **C2** against the tested cancer cell lines appears to be the result of cell cycle arrest in the sub-G1 phase (HepG2 cells) or G1/M phase (T98G cells) associated with DNA damage. Nevertheless, more work is needed to clarify the molecular mechanism of action of compound **C2** leading to apoptosis. 

#### 2.4.4. Antimicrobial Activity

The in vitro antimicrobial activities of three ligands, **L2**–**4**, and their complexes, **C2**–**4**, were investigated on reference strains of Gram-positive bacteria, namely *Staphylococcus aureus* ATCC 25923, *Staphylococcus aureus* ATCC BAA-1707, *Staphylococcus epidermidis* ATCC 12228, *Micrococcus luteus* ATCC 10240 and *Bacillus cereus* ATCC 10876 and Gram-negative bacterial strains of *Salmonella Typhimurium* ATCC 14028, *Escherichia coli* ATCC 25922, *Proteus mirabilis* ATCC 12453, *Klebsiella pneumoniae* ATCC 13883 and *Pseudomonas aeruginosa* ATCC 9027, as well as three strains of yeasts such as *Candida albicans* ATCC 102231, *Candida parapsilosis* ATCC 22019 and *Candida glabrata* ATCC 90030.

The tested compounds did not show antibacterial activity against Gram-positive nor Gram-negative bacteria (MIC > 1000 mg/L,). As revealed by the data in Table 4, they displayed mild or no bioactivity towards the yeasts that were tested, except for ligand **L3**, which demonstrated moderate anti-*Candida* activity (MIC = 125–250 mg/L).

Considering the promising anticancer potential of the tested complexes, it is notable that no antimicrobial activity is a beneficial property of these complexes as they do not cause harm to human microbiota during treatment. The gut microbiota plays a significant role in maintaining normal gut physiology and body health. It includes protection from pathogens by colonizing mucosal surfaces, producing different antimicrobial substances and enhancing the immune system, playing a significant role in digestion and metabolism, as well as influencing brain–gut communication and thus impacting the mental and neurological functions of the host [84]. 

### 2.5. Determination of Free Radical Scavenging Capacity

The disturbed redox balance between reactive oxygen species (ROS) and the antioxidant system is a critical factor in cancer development. One of the strategies for reducing tumors is targeting the redox metabolism by increasing the antioxidant capacity of cancer cells. In this way, antioxidants are being studied to develop more effective anticancer therapy [85]. Furthermore, it has been reported that some oxidants may act as the inducers of DNA damage response, which may result in cell death [86,87].

The free radical scavenging abilities of the free ligands, **L1**, **L2**, **L3** and **L4**, and the corresponding copper(II) complexes, **C1**, **C2**, **C3** and **C4**, were analyzed with two colorimetric methods, DPPH (2,2′-diphenyl-1-picrylhydrazyl) and ABTS (2,2′-azinobis(3-ethylbenzothiazoline-6-sulphonic acid) assays, which were previously used to study the antioxidant activities of the Cu(II) complexes [88]. The results are expressed as IC_50_—the sufficient concentration to obtain 50% of the maximum scavenging activity—and they are shown in Table 5. Ascorbic acid, a well-known antioxidant, was used as a positive control. 

From our results, copper(II) complexes **C1**–**4** exhibited moderate to good DPPH scavenging ability compared with the ascorbic acid (IC_50_ = 26.46–401.52 µg/mL vs. IC_50_ = 11.65 µg/mL), while ligands **L1**–**4** showed no activity in the DPPH assay; they did not reach the IC_50_ value despite the increasing concentration until 2 mg/mL (higher concentrations resulted in precipitation). The highest DPPH antiradical activity was found for Cu(II) complex **C4** containing 3-ethylimidazolidin-2-one moiety (R = C_2_H_5_, IC_50_ = 26.46 µg/mL). The Cu(II) complex **C1** bearing an azetidin-2-one ring system was observed to display slightly weaker activity (IC_50_ = 37.45 µg/mL). In turn, the analogue of **C4** with 3-methylimidazolidin-2-one functionality (complex **C3**, R = CH_3_) showed a significant decrease in potency (IC_50_ = 380.65 µg/mL). A further decrease in the radical scavenging capability was observed for complex **C2** (IC_50_ = 401.52 µg/mL) with imidazolidin-2-one scaffold (R = H). 

On the other hand, in the ABTS assay, all tested compounds displayed antioxidant capacity with IC_50_ values in the range from 72.5 µg/mL to 183.21 µg/mL (Table 5). The free ligands **L2** and **L3** demonstrated slightly higher ABTS quenching ability when compared to their complexes **C2** and **C3** (IC_50_ = 82.08 and 96.76 µg/mL vs. IC_50_ = 107.14 and 106.19 µg/mL, respectively), while ligands **L1** and **L4** proved to be less potent than the corresponding complexes **C1** and **C4** (IC_50_ = 183.21 and 108.59 µg/mL vs. IC_50_ = 112.67 and 72.7 µg/mL, respectively). As in the DPPH assay, the most promising antiradical properties for ABST radical scavenging ability were presented by complex **C4** (IC_50_ = 72.7 µg/mL).

It could be concluded that in the DPPH assay, the coordination of ligands to the copper(II) metal center appears to be beneficial for antiradical potency as was previously reported [88,89,90]. In general, no similar correlation was found for complexes **C1**–**4** compared with their ligands **L1**–**4** in the ABTS analysis. Nevertheless, it should be pointed out that the highest scavenging activity on both the DPPH and ABTS radicals was exhibited by Cu(II) complex **C4**. This observation suggests that the presence of the electron-donating ethyl group at the N-3 position of the imidazolidin-2-one moiety (R = C_2_H_5_) facilitates antioxidant activity in the complex **C4** by increasing the electron density at the central ion, leading to improved radical scavenging ability in the molecule [91]. However, it was not possible to derive a correlation between antioxidant and antiproliferative activities with the only exception of Cu(II) complex **C4**, which demonstrated remarkable activity on the cancer cell lines, especially HepG2, LS180 and T98G (IC_50_ = 5.92–9.27 µg/mL), and the strongest antioxidant properties within the tested group (IC_50_ = 26.46 µg/mL in DDPH and 72.7 µg/mL in ABTS). On the contrary, the most potent complex against cancer cells, copper(II) complex **C2** (IC_50_ = 5.04–6.97 µg/mL), exhibited less antiradical potency (IC_50_ = 401.52 µg/mL in DDPH, and 107.13 µg/mL in ABTS). 

It is worth noting that due to the redox activity of the copper(II) complexes, some of the previously reported copper(II) compounds combine both antioxidant and pro-oxidant modes of action, inducing apoptosis in tumor cells [87]. However, regarding the results of our studies, further work will be needed to clarify this.

### 2.6. In Silico Physicochemical, Pharmacokinetic and Drug-Likeness Predictions

The basic features of a drug molecule that determine whether it can be absorbed and transported inside the body include its solubility, lipophilicity, charge and size. Lipinski’s rules dictate that undissociated substances with molecular weights below 500 Da, and a lipophilicity level in the range of 1–3 will have the best absorption rate. 

**Figure 13:**
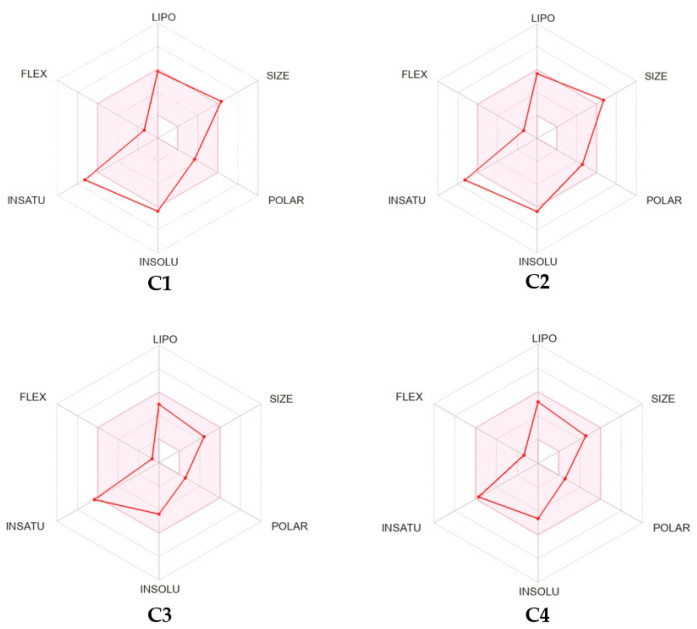
Oral bioavailability radar charts for the studied compounds **C1**, **C2**, **C3** and **C4**. In bioavailability radar, the pink area represents the optimal range for each physicochemical property of oral bioavailability, while the red lines represent compounds **C1**, **C2**, **C3** and **C4** (LIPO—lipophilicity SIZE—size; POLAR—polarity; INSOLU—solubility; INSATU—saturation; FLEX—flexibility).

**Figure 14:**
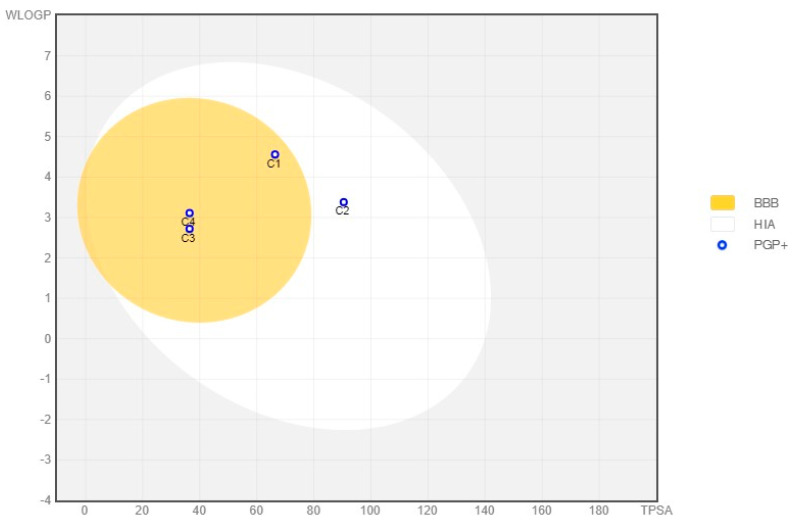
BOILED−egg plot for the studied copper(II) complexes **C1**, **C2**, **C3** and **C4**.

The estimation of drug likeliness and the prediction of the physicochemical and pharmacokinetic properties—ADME (absorption, distribution, metabolism and excretion)—of copper(II) complexes **C1**, **C2**, **C3** and **C4** were carried out using the free available web tool SwissADME [92]. The prediction of the principal properties of the molecules was carried out by using Lipinski’s filter, which confirmed the drug likeness of the synthesized copper(II) complexes. The results of the calculated basic parameters of Cu(II) complexes **C1**, **C2**, **C3** and **C4** are presented in Table 6 and Figure 13 and Figure 14 (for more details, see Table S4 in the Supplementary Materials). 

The topological polar surface area (TPSA) of a molecule can be defined as the sum of the polar atoms, namely oxygen and nitrogen, as well as their hydrogen atoms attached. A heightened TPSA rate (>140 Å^2^) may be attributed to poor membrane permeability and blood–brain barrier accessibility. Thus, it can be said that a TPSA is a metric for describing the ability of compounds to permeate living cells [93]. 

As can be seen from the data in Table 6, copper(II) complexes **C1**–**4** are characterized by reasonable polarity. Their TPSA values are in the range of 33.20–66.40 Å^2^, except for compound **C4**, which has an estimated value equaling 90.46 Å^2^. All complexes possess a suitable lipophilicity estimated as a partition coefficient between *n*-octanol and water, with consensus logP (ClogP) values ranging from 1.87 to 3.04. Moreover, three copper(II) complexes, **C1**, **C3** and **C4**, are expected to be moderately soluble in water. 

Lipinski’s rule of five indicates that the lead compound should not contain more than 5 hydrogen bond donors (HBD), while hydrogen bond acceptors (HBAs) should not exceed 10. The calculated copper(II) complexes **C1**–**4** exhibited two or four HBAs, and the aforementioned standard was congregated. According to the Veber’s rule (rotatable bonds must be equivalent to 10 and PSA must be lower than 140 Å), the complexes are also expected to possess high oral bioavailability.

Moreover, according to Table 6, the bioavailable radar charts in Figure 13 and the “BOILED-egg” plot in Figure 14, the investigated copper(II) complexes **C1**, **C2**, **C3** and **C4** are predicted to possess high gastrointestinal tract (GI) absorption and blood–brain barrier BBB permeant. In this regard, all of the tested molecules show the same bioavailability score of 0.55, which suggests desirable pharmacokinetic properties. 

In light of Lipinski’s “rule of five”, copper(II) complexes **C1** and **C2** slightly exceed the molecular weight and violate this criterion, while copper(II) complexes **C3** and **C4** meet all of the criteria as one of the key drug-likeness characteristics. Furthermore, according to Table 6, the ADME properties of copper(II) complexes are favorable and indicate that designed compounds may be considered drug-likeness molecules.

## 3. Materials and Methods 

### 3.1. Chemistry

#### 3.1.1. General Information

Melting points were determined with a Boëtius apparatus and are uncorrected. The infrared spectra were obtained on KBr pellets using a Nicolet 380 FT-IR 1600 spectrophotometer (Thermo Fisher Scientific Inc., Waltham, MA, USA). The elemental analyses for C, H and N were within 0.4% of the theoretical values. Thin layer chromatography (TLC) of ligands **L1**, **L2**, **L3** and **L4** was performed on silica gel precoated 60 F254 Merck plates (Merck KGaA, Darmstadt, Germany) using the following eluents: chloroform and ethyl acetate (8:2, *v*/*v*), chloroform and methanol (99:1, *v*/*v*) or chloroform, ethyl acetate and methanol (8:1.5:0.5, *v*/*v*/*v*). The developed chromatograms were viewed under UV light at 254 nm.

The mass spectrum of ligand **L4** was recorded on a Shimadzu LCMS-2010 EV (Tokyo, Japan) spectrometer equipped with an electrospray source, and the ESI-MS spectrum was registered in positive ion mode. 

The difference spectra of copper complexes **C1**, **C2**, **C3** and **C4** from λ = 250 nm to 600 nm were recorded at 37 °C with a UV-VIS spectral photometer, Specord S600 (Analytik Jena, Jena, Germany), over the course of 3 h, with spectra being recorded every 10 min. Copper(II) complexes were dissolved in dimethylformamide (DMF) (Sigma-Aldrich Chemie GmbH, Steinheim, Germany). Freshly prepared solution of copper(II) complex at concentration 4 mM (25 µL) was added to 2.475 mL of 100 mM phosphate-buffered solution (pH 7.4), giving the complex a final concentration of 40 µM. Baseline correction was carried out by subtracting the mean absorption between λ = 250 and 600 nm from each spectrum. 

The ligands **L1** and **L3**, and the new ligand **L4**, were prepared as previously reported [64]. Ligand **L2** was synthesized using a modified method [69].

#### 3.1.2. Synthesis of 1-(Isoquinolin-3-yl)imidazolidin-2-one (**L2**)

Isoquinoline 2-oxide (0.726 g, 0.005 mol) was added in one portion to freshly prepared solution of 2-chloro-4,5-dihydro-1*H*-imidazole (2.5 g, 0.025 mol) in chloroform (30 mL) at room temperature (20–22 °C) [94]. The stirring was continued until the exothermic reaction subsided (30–60 min.), and then the mixture was cooled. The precipitated crude product was isolated by suction, washed with chloroform (2 × 5 mL) and made alkaline with aqueous 20% potassium carbonate. Then, the oily residue was extracted with chloroform (3 × 20 mL). The collected organic layers were dried with anhydrous magnesium sulfate, filtered and concentrated to dryness. Upon addition of acetone to the oily residue, the product **L2** was precipitated, collected by filtration and washed with acetone. Ligand **L2** was purified on silica gel using preparative thin-layer chromatography (chromatotron); eluent: chloroform/ethyl acetate (4:1, *v*/*v*), yield: 43%, m.p. 234–236 °C (m.p. 233–236 °C [64]); IR (KBr) *v* (cm^−1^): 3201, 3111, 3053, 2996, 2965, 2902, 1717, 1625, 1583, 1488, 1454, 1415, 1365, 1268, 877, 750. Calculated for C_12_H_11_N_3_O (213.24): C, 67.59; H, 5.20; N, 19.71. Found: C, 67.78; H, 5.08; N, 19.96. 

#### 3.1.3. Synthesis of 1-Ethyl-3-(isoquinolin-3-yl)imidazolidin-2-one (**L4**) 

To a stirred suspension of ligand **L2** (0.213 g, 1 mmol) in 1–2 mL of anhydrous DMF, solid NaOH (0.1 g, 2.5 mmol) and ethyl iodide (0.9358 g, 0.4823 mL, 6 mmol) were added. After 48 h, a mixture was dissolved with chloroform (15 mL) and evaporated to dryness. Compound **L4** was separated using preparative thin-layer chromatography (chromatotron); eluent: dichloromethane/ethyl acetate/methanol, (8:1.5:0.5, *v*/*v*/*v*); yield: 62%; m.p. 151–153 °C; IR (KBr) *v* (cm^−1^): 3114, 3059, 2970, 2922, 2890, 1688, 1626, 1582, 1488, 1426, 1389, 1360, 1270, 878; ^1^H-NMR (DMSO-*d_6_*, 400 MHz) *δ* (ppm): 1.12 (t, 3H, CH_3_), 3.30 (q, 2H, CH_2_), 3.47–3.51 (m, 2H, CH_2_), 4.04–4.08 (m, 2H, CH_2_), 7.47 (t, 1H, CH^7^-isoquin.), 7.66 (t, 1H, CH^6^-isoquin.), 7.83 (d, *J* = 8.3 Hz, 1H, CH^5^-isoquin.), 8.00 (dd, *J*_1_ = 1.2 Hz, *J*_2_ = 8.3 Hz, 1H, CH^8^-isoquin.), 8.48 (s, 1H, CH^4^-isoquin.), 9.12 (s, 1H, CH^1^-isoquin.); ^13^C-NMR (DMSO-*d_6_*, 100 MHz) *δ* (ppm): 12.81, 38.40, 40.97, 41.77, 105.36, 125.19, 125.39, 126.50, 127.96, 131.11, 137.53, 148.63, 151.11, 157.00; MS (ESI, CH_3_OH:CH_3_CN+0,1% CH_3_COOH, 1:1, *v*/*v*) *m*/*z* = 242 [M+H]^+^, *m*/*z* = 264 [M + Na]^+^, and *m*/*z* = 305 [M + Na + CH_3_CN]^+^. Calculated for C_14_H_15_N_3_O (241.29): C, 69.69; H, 6.27; N, 17.41. Found: C, 69.51; H, 6.02; N, 17.72.

#### 3.1.4. Synthesis of Copper(II) Complexes of 1-(Isoquinolin-3-yl)heteroalkyl-2-ones **C1**–**4** (General Method)

To a solution of appropriate ligand (**L1**–**4**) in 99.5% DMF (2–3 mL) at a temperature of 60–70 °C, copper(II) chloride dihydrate (CuCl_2_^.^2H_2_O) dissolved in 1 mL of DMF was added dropwise in a molar ratio of 1:1 (**C3**, and **C4**) or 2:1 (**C1**, **C2**). The mixture was then left at ambient temperature (20–22 °C) for slow evaporation of the solvent (3–12 days). The green precipitate of the copper(II) complex was filtered off, washed with a small amount of 99.5% dimethylformamide and dried. Using the above procedure, the following copper(II) complexes were obtained: 

##### Dichloro{bis[1-(Isoquinolin-3-yl)azetidin-2-one]}copper(II) (**C1**)

For the reaction, 0.132 g (0.664 mmol) of 1-(isoquinolin-3-yl)azetidin-2-one (**L1**) and 0.057 g (0.332 mol) copper(II) chloride dihydrate were used. After 12 days, 0.048 g of copper(II) complex **C1** was obtained, yield 27%, m.p. 188–190 °C; **IR** (KBr) *v* (cm^−1^): 3110, 3060, 3031, 2959, 2894, 1751, 1630, 1594, 1472, 1387, 1287, 1247, 1154, 1087, 1027, 968, 883, 775, 464. Calculated for C_24_H_20_Cl_2_CuN_4_O_2_ (530.89): C, 54.30; H, 3.80; N, 10.55. Found: C, 54.05; H, 3.97; N, 10.40.

##### Dichloro{bis[1-(Isoquinolin-3-yl)imidazolidin-2-one]}copper(II) (**C2**)

For the reaction, 0.15 g (0.702 mmol) of 1-(isoquinolin-3-yl)imidazolidin-2-one (**L2**) and 0.06 g (0.351 mol) copper(II) chloride dihydrate were used. After 10 days, 0.052 g of copper(II) complex **C2** was obtained, yield 33%, m.p. > 350 °C (decomp.); **IR** (KBr) *v* (cm^−1^): 3106, 3022, 2827, 1670, 1633, 1596, 1464, 1434, 1368, 1285, 1149, 757, 657, 564, 476. Calculated for C_24_H_22_Cl_2_CuN_6_O_2_ (560.92): C, 51.39; H, 3.95; N, 14.98. Found: C, 51.12; H, 3.84; N, 14.71. Found: C, 52.25; H, 4.05; N, 14.65. 

##### Dichloro[1-(Isoquinolin-3-yl)-3-methylimidazolidin-2-one]copper(II) (**C3**)

For the reaction, 0.09 g (0.396 mmol) of 1-(isoquinolin-3-yl)-3-methylimidazolidin-2-one (**L3**) and 0.068 g (0.396 mol) copper(II) chloride dihydrate were used. After 3 days, 0.045 g of copper(II) complex **C4** was obtained, yield 61%, m.p. 315–316 °C; **IR** (KBr) *v* (cm^−1^): 3464, 3389, 3072, 2947, 2891, 1655, 1635, 1596, 1519, 1465, 1410, 1396, 1294, 1286, 757, 466. Calculated for C_13_H_13_Cl_2_CuN_3_O (361.71): C, 43.17; H, 3.62; N, 11.62. Found: C, 43.11; H, 3.57; N, 11.23. 

##### Dichloro[1-ethyl-3-(isoquinolin-3-yl)imidazolidin-2-one]copper(II) (**C4**)

For the reaction, 0.09 g (0.373 mmol) of 1-ethyl-3-(isoquinolin-3-yl)imidazolidin-2-one (**L4**) and 0.064 g (0.373 mol) copper(II) chloride dihydrate were used. After 5 days, 0.1 g of copper(II) complex **C5** was obtained, yield 68%, m.p. 270–274 °C; **IR** (KBr) *v* (cm^−1^): 3043, 2967, 2923, 2852, 1639, 1518, 1458, 1289, 732, 460. Calculated for C_14_H_15_Cl_2_CuN_3_O (375.74): C, 44.75; H, 4.02; N, 11.18. Found: C, 44.64; H, 4.22; N, 11.34. Found: C, 43.11; H, 3.57; N, 11.23.

#### 3.1.5. Single Crystal X-ray Diffraction Studies

Single crystals of copper(II) complex **C1** suitable for X-ray diffraction were obtained by slow solvent evaporation at room temperature from DMF. Diffraction measurements were performed using an XtaLAB Synergy, Dualflex diffractometer (CrysAlisPro (Rigaku Oxford Diffraction, Tokyo, Japan, 2023) with a Pilatus 300 K detector at low temperature (100.0(2) K) using MoKα radiation for complex **C1**. Diffraction data were processed using CrysAlisPRO (version 1.171.42.86a) software (Rigaku Oxford Diffraction, 2023). Solving and refinement of the crystal structure were performed with SHELX (version 2018/2) [95] and SHELXL (version 2019/3) [96] using full-matrix least-squares minimization on F^2^. All H atoms were optimized using constraints with riding model and distances suitable for 100 K temperature and with U_iso_(H) = 1.2 U_eq_(C). ShelXle (version 1143) software [97] was used to visualize the molecular structure. Graphical representation of the crystal structures was performed using the Mercury program (version 2022.3.0) [98]. OLEX2 program [99] was used in data preparation. The tables were prepared using PublCIF software (version 1.9.21_c) [100]. 

### 3.2. Aqueous Stability Studies

The stability of copper(II) complexes **C1**, **C2**, **C3** and **C4** was determined in 100 mM phosphate-buffered solution (pH 7.4) at 37 °C by using a Specord S600 (Analytic Jena, Jena, Germany) UV-vis diode-array photometer. Difference spectra were recorded every 10 min to help identify very small changes in the UV-vis spectra over 3 h incubations. 

### 3.3. Biological Studies

#### 3.3.1. Examination of the Cytotoxic Activity with Assessment by MTT Assay

Cytotoxic activity of the investigated compounds was evaluated against T98G (glioblastoma), HepG2 (hepatoma), LS-180 (colon cancer) and A375 (melanoma) cell lines. Human normal skin fibroblasts, CCD-1059Sk (CRL-2072), were used as reference (non-cancerous) cells. All of the cell lines were obtained from American Type Culture Collection (ATCC; Manassas, VA, USA). A375, T98G and CCD-1059Sk cells were cultured in Dulbecco’s Modified Eagle’s Medium (DMEM) (Sigma Aldrich, St. Louis, MO, USA) supplemented with 10% FBS, penicillin (100 U/mL) and streptomycin (100 μg/mL). LS-180 and HepG2 cells were cultured in Eagle’s Minimum Essential Medium supplemented with 10% FBS, penicillin (100 U/mL) and streptomycin (100 μg/mL). All of the cells were maintained at 37 °C in a humidified atmosphere of 5% CO_2_. The compounds were dissolved in DMSO in order to obtain stock solutions. At the day of the experiment, the suspension of cells (1 × 10^5^ cells/mL) was distributed onto 96-well plates at a volume of 100 μL/well. After attachment, the cells were treated with the increased concentrations of the tested compounds in medium containing 2% FBS and incubated for 24 h. Then, the medium was removed from wells, and cells were rinsed with PBS. Afterwards, 15 μL of MTT working solution (5 mg/mL in PBS) was added to each well, and the plates were incubated for 3 h. Subsequently, 100 μL of 10% SDS solution was added to each well in order to dissolve the precipitated formazan crystals. After overnight incubation at 37 °C, the absorbance of the obtained solution was measured at λ = 570 nm using a microplate reader (Epoch, BioTek Instruments, Inc., Winooski, VT, USA). At least two independent experiments were performed in triplicate. DMSO in the concentrations present in the dilutions of stock solutions did not influence the viability of the tested cells. IC_50_ values of the investigated derivatives and positive control (*etoposide*) were calculated using the IC_50_ online calculator [101]. 

#### 3.3.2. Cell Cycle Analysis

Cell cycle analysis of T98G and HepG2 cells pretreated with copper(II) complex **C2** (**CX**) (at IC_50_ concentration) was performed on NucleoCounter NC-3000 Image Cytometer (ChemoMetec, Lillerod, Denmark). The investigated cells were seeded on 6-well culture plates (Corning Inc., New York, NY, USA) at the density of 1 × 10^5^ cells/mL and cultured in the respective medium at 37 °C in a humidified atmosphere of 5% CO_2_. When the cells reached approximately 80% confluence, they were treated with **C2** (**CX**), at a concentration of IC_50_, for 24 h. Subsequently, the cells were detached with trypsine, suspended in 250 μL of lysis buffer (Solution 10) supplemented with DAPI (10 μg/mL) and incubated for 5 min at 37 °C. Following this, stabilization buffer (Solution 11) was added, and the obtained cell suspension was applied on NC-slide and analyzed using NucleoCounter NC-3000 Image Cytometer equipped with NucleoView NC-3000^TM^ software (ChemoMetec A/S, Lillerod, Denmark). Experiments were repeated three times, and the measurements in each experiment were run in duplicate.

#### 3.3.3. Analysis of Interaction of Copper(II) Complex **C2** (**CX**) with Clinically Used Anticancer Agents 

Possible interactions between copper(II) complex **C2** (**CX**) and anticancer drugs including *etoposide* (**ETO**), *cisplatin* (**CIS**), *5-fluorouracil* (**5-FU**) and *temozolomide* (**TMZ**) were examined on the most sensitive cancer cell lines, i.e., T98G and HepG2. Firstly, the highest concentrations of **C2** (**CX**) that did not affect the viability of T98G and HepG2 cells, as well as the IC_50_ values for **ETO**, **CIS**, **5-FU** and **TMZ**, were established using MTT assay (as described above). Chemotherapeutics whose IC_50_ values were higher than 100 µg/mL were tested at the maximal concentration of 100 µg/mL. Next, T98G or HepG2 cells were incubated for 24 h with medium containing a mixture of the respective drugs mixed with the highest non-toxic concentration of **C2** (**CX**). The viability of cells was evaluated using MTT assay, as described above. ANOVA analysis (with Tukey’s post hoc test) was performed in order to examine the possible interactions between compound **C2** (**CX**) and **ETO**, **CIS**, **5-FU** and **TMZ**. 

#### 3.3.4. In Vitro Antimicrobial Activity

Antibacterial and antifungal activities of the free ligands **L2**, **L3** and **L4** and the corresponding copper(II) complexes **C2**, **C3** and **C4** were screened using the two-fold microdilution broth method. Minimal inhibitory concentrations (MICs) of tested compounds for the panel of reference Gram-positive bacteria, including *Staphylococcus aureus* ATCC 25923, *Staphylococcus aureus* ATCC BAA-1707, *Staphylococcus epidermidis* ATCC 12228, *Micrococcus luteus* ATCC 10240 and *Bacillus cereus* ATCC 10876; Gram-negative bacteria, including *Salmonella Typhimurium* ATCC 14028, *Escherichia coli* ATCC 25922, *Proteus mirabilis* ATCC 12453, *Klebsiella pneumoniae* ATCC 13883 and *Pseudomonas aeruginosa* ATCC 9027; and yeasts, including *Candida albicans* ATCC 102231, *Candida parapsilosis* ATCC 22019 and *Candida glabrata* ATCC 90030 were determined. The procedure has been described in detail before [102]. Briefly, the solutions of tested compounds dissolved in dimethylosulfoxide (DMSO) were suspended in Mueller–Hinton broth for bacteria or Mueller–Hinton broth with 2% glucose for fungi. Then, the series of two-fold dilutions were carried out in the sterile Nunc™ MicroWell™ 96-Well Microplates (ThermoFisher Scientific Inc.), obtaining concentrations from 1000 to 7.8 mg/L in the appropriate medium. Simultaneously, the inocula of 24 h cultures of microorganisms in sterile physiological saline (0.5 McFarland standard density) were prepared and added to each well, obtaining final density of 5 × 10^5^ CFU/mL for bacteria and 5 × 10^4^ CFU/mL for yeasts. Proper positive (inoculum without tested compound) and negative (compound without inoculum) controls were added in each microplate. After incubation (35 °C, 24 h), the growth of microorganisms was measured spectrophotometrically at 600 nm (BioTEK ELx808, Bio-Tek Instruments, Inc., Winooski, VT, USA). MICs were marked at the lowest concentration of the compound without the growth of bacteria or fungi.

### 3.4. Antioxidant

#### 3.4.1. Materials

Ascorbic acid, oleanolic acid, DPPH (2,2-diphenyl-1-picrylhydrazyl), ABTS (2,2′-azino-bis(3-ethylbenzothiazoline-6-sulfonic acid) diammonium salt, potassium persulfate and DMSO (dimethyl sulfoxide) were sourced from Sigma Chemical Co. (St. Louis, MO, USA). TRIS-HCl (0.2 M, pH 8) and HPLC-grade methanol were sourced from P.O.Ch. (Gliwice, Poland).

#### 3.4.2. DPPH Assay 

The DPPH radical scavenging assay of samples was performed with ascorbic acid as a positive control [88]. Briefly, 100 μL of different concentrations of the complexes, dissolved in DMSO, was mixed with 100 μL of 0.06 mM DPPH methanolic solution and incubated at room temperature in the dark for 30 min. The change in absorbance at λ = 517 nm was analyzed with the use of a 96-well microplate reader (Epoch, BioTek System, Winooski, VT, USA). The control was composed of DPPH and DMSO. 

DPPH inhibition was calculated according to the following equation:

DPPH Inhibition (%) = [(Acontrol − Asample)/Acontrol] × 100%

The radical scavenging activity of the samples was shown as the IC_50_ value (the concentration of the analyzed samples that caused a decrease in the non-reduced form of the DPPH radical by 50%).

#### 3.4.3. ABTS Assay

The ABTS radical scavenging assay of samples (ligands or complexes) was performed with ascorbic acid as a positive control [88]. Briefly, 30 μL of different concentrations of the samples, dissolved in DMSO, was mixed with 170 μL of ABTS solution (2 mM ABTS diammonium salt, 3.5 mM potassium persulfate) and completed with water to a final volume of 300 μL. ABTS solution with DMSO was used as a control. After 10 min of incubation at 30 °C in the dark, a change in absorbance was observed at λ = 750 nm by a 96-well microplate reader (Epoch, BioTek System, Santa Clara, CA, USA). 

ABTS inhibition was calculated according to the following equation:

ABTS Inhibition (%) = [(Acontrol − Asample)/Acontrol] × 100%

The radical scavenging activity of the samples was shown as the IC_50_ value (the concentration of the analyzed samples that caused a decrease in the non-reduced form of the ABTS radical by 50%). 

### 3.5. ADME/Drug-Likeness Calculation

The physicochemical, pharmacokinetic and drug-likeness properties of copper(II) complexes **C1**–**4** were predicted using the SwissADME web tool, which is available online [103]. 

## 4. Conclusions

Four new copper(II) complexes, **C1**–**4**, derived from 1-(isoquinolin-3-yl)heteroalkyl-2-one ligands, **L1**–**4**, were prepared, and their coordination modes were established using an elemental analysis, infrared spectra as well as X-ray crystallographic study for **C1**. The structures of copper(II) complexes **C1** and **C2** were determined as mononuclear species incorporating two molecules of the neutral 1-(isoquinolin-3-yl)heteroalkyl-2-one ligand bound to the central copper ion via a bidentate manner. Interestingly, ligands **L3** and **L4** containing an alkyl group at the N-3 position of the imidazolidin-2-one moiety form tetra-coordinate mononuclear copper(II) complexes **C3** and **C4** with the central atom chelated by one molecule of the neutral bidentate ligand.

The results of UV-vis spectrophotometry confirmed the stability of complexes **C1**–**4** under conditions that mimic the physiological environment.

The in vitro cytotoxicity studies showed that the coordination of 1-(isoquinolin-3-yl)heteroalkyl-2-one ligands **L1**–**4** with a copper(II) ion results in metal complexes **C1**–**4** with remarkable growth inhibitory properties against tested human cancer cell lines, especially hepatoma HepG2, colon cancer LS-180 and glioblastoma T98G cells. The cytotoxic effect of the synthesized copper(II) complexes towards HepG2, LS-180 and T98G cancer cells was higher than the known antitumor agent *etoposide*. Among these compounds, dichloro{bis[1-(isoquinolin-3-yl)imidazolidin-2-one]}copper(II) (**C2**) was found to be the most promising agent with the greatest selective effect on HepG2, LS180 and T98G cancer cell lines compared with the non-cancerous CCD-1059-Sk cell line. The complex **C2** induced sub-G1 cell cycle arrest in the HepG2 cells and induced G1/M cell cycle arrest in the T98G cells, which was accompanied by DNA degradation. Furthermore, the treatment of HepG2 and T98G cells with the tested copper(II) compound **C2** at the concentration that did not inhibit the growth of cancer cells resulted in a significant increase in the cytotoxic effects of chemotherapeutics such as *etoposide*, *5-fluorouracil* and *temozolomide.* To clarify the mechanism of synergism between **C2** and the clinically used drugs, more advanced studies are needed. 

In turn, in microbiological tests, the investigated 1-(isoquinolin-3-yl)heteroalkyl-2-one ligands **L2**–**4** and their copper(II) complexes **C2**–**4** were inactive, except for a 1-(isoquinolin-3-yl)-3-methylimidazolidin-2-one ligand (**L3**), which exhibited moderate anti-*Candida* activity. 

The antioxidant activity results suggest that in the DPPH test, the coordination of ligands **L1**–**4** with a copper(II) metal center is beneficial for antiradical potency. No direct correlation was found between antiproliferative and antioxidant effects; the exception to this was dichloro[1-ethyl-3-(isoquinolin-3-yl)imidazolidin-2-one]copper(II) (**C4**), which demonstrated remarkable growth-inhibitory properties against cancer cells and the strongest antioxidant activity in both the DPPH and ABTS assays within the tested compounds. On the other hand, the copper(II) compound **C4**, which had the highest potency against the tested tumor cell lines, exhibited moderate antiradical properties.

Generally, the prediction of ADME/drug-likeness properties revealed that the tested copper(II) complexes may be considered as drug-likeness molecules.

In summary, the results obtained may be useful as a starting point for the development of novel copper-based antitumor agents. 

## Figures and Tables

**Table 1:** X-ray diffraction data and structure refinement details for copper(II) complex **C1**.

Compound	(C1_1_auto)
Chemical formula	C_24_H_20_Cl_2_CuN_4_O_2_
*M*_r_	530.89
Crystal system, space group	Triclinic, $P\bar{1}$
Temperature (K)	100
*a*, *b*, *c* (Å)	7.8288 (2), 8.2173 (2), 9.5081 (2)
α, β, γ (°)	106.092 (2), 111.334 (3), 97.897 (2)
*V* (Å^3^)	527.68 (2)
*Z*	1
Radiation type	Mo *K*α
μ (mm^−1^)	1.32
Crystal size (mm)	0.22 × 0.2 × 0.09
Diffractometer	XtaLAB Synergy, Dualflex, Pilatus 300 K
*T*_min_, *T*_max_	0.586, 1.000
No. of measured, independent and observed [*I* > 2σ(*I*)] reflections	15,833, 3057, 2752
*R*_int_	0.040
(sin θ/λ)_max_ (Å^−1^)	0.748
*R*[*F*^2^ > 2σ(*F*^2^)], *wR*(*F*^2^), *S*	0.029, 0.072, 1.09
No. of reflections	3057
No. of parameters	151
H-atom treatment	H-atom parameters constrained
Δ_max_, Δ_min_ (e Å^−3^)	0.91, −0.35

**Table 2:** Hydrogen bond geometry for copper(II) complex **C1**.

*D*—H···*A*	*D*—H (Å)	H⋯*A* (Å)	*D*···*A* (Å)	*D*—H⋯*A* (*º*)
C9—H9···Cl1 ^i^	0.95	2.86	3.7205 (15)	152
C2—H2···O1 ^ii^	0.95	2.21	2.9512 (19)	135

Symmetry codes: ^i^ *x*, *y* + 1, *z*; ^ii^ −*x* + 1, −*y*, −*z* + 1.

**Table 3:** Cytotoxic activity of the investigated free ligands **L1**–**4**, Cu(II) complexes **C1**–**4** and reference compound against human cancer cell lines and non-cancerous cells determined by MTT assay after 24 h incubation.

Ligands/Complexes	IC_50_ ± SD (μg/mL)				
	Cell Line				
	A375	HepG2	LS180	T98G	CCD-1059-Sk
**L1**	>200	>200	>200	>200	>200
**C1**	37.97 ± 3.01	14.89 ± 0.56	11.46 ± 0.69	8.25 ± 0.36	18.83 ± 0.91
**L2**	>200	>200	>200	>200	>200
**C2**	28.91 ± 1.79	5.04 ± 0.42	5.88 ± 0.40	6.97 ± 0.52	21.86 ± 1.03
**L3**	>200	>200	>200	>200	>200
**C3**	22.78 ± 1.14	12.00 ± 0.36	7.09 ± 0.53	10.55 ± 0.74	25.17 ± 0.85
**L4**	>200	>200	>200	>200	>200
**C4**	37.80 ± 3.11	6.72. ± 0.12	5.92 ± 0.54	9.27 ± 0.11	18.25 ± 1.37
Etoposide *	10.20 ± 0.83	43.21 ± 2.75	>100	>100	83.53 ± 3.19

* *Etoposide* was used as a positive control; IC_50_—the concentration that inhibits 50% of cell viability. The values shown are mean ± SD from three repetitions in two independent experiments.

**Table 4:** Antimicrobial activity of free ligands, **L2**, **L3** and **L4**, and their complexes, **C2**, **C3** and **C4**, against reference strains of yeasts.

Microorganism/Compounds	L2	C2	L3	C3	L4	C4
	MIC (mg/L) *					
Yeasts						
*C. albicans* ATCC 102231	500	1000	250	1000	1000	500
*C. parapsilosis* ATCC 22019	1000	1000	125	1000	>1000	>1000
*C. glabrata* ATCC 90030	>1000	>1000	250	1000	1000	>1000

* MIC—minimum inhibitory concentration in milligrams per liter.

**Table 5:** Antiradical activity (DPPH and ABTS) of ligands **L1**, **L2**, **L3** and **L4** and their copper(II) complexes **C1**, **C2**, **C3** and **C4** expressed as IC_50_ (µg/mL) with standard deviation (±SD). Ascorbic acid was used as a positive control.

Ligands/ Complexes	DDPH	ABTS
**L1**	NR *	183.21 ± 2.45
**C1**	37.45 ± 0.66	112.67 ± 1.8
**L2**	NR *	82.08 ± 2.77
**C2**	401.52 ± 2.48	107.14 ± 1.42
**L3**	NR *	96.67 ± 2.84
**C3**	380.65 ± 2.74	106.19 ± 2.55
**L4**	NR *	108.59 ± 1.51
**C4**	26.46 ± 1.04	72.5 ± 0.97
**Ascorbic acid**	11.65 ± 0.54	20.15 ± 0.33

* NR—the IC_50_ value was not reached.

**Table 6:** Predicted physicochemical, pharmacokinetic and drug-likeness properties of copper(II) complexes **C1**, **C2**, **C3** and **C4**.

	Physicochemical Properties					Lipophilicity	Water Solubility	Pharmacokinetics		Drug Likeness	
	mol. wt. (g/mol)	ROTB (n)	HBA (n)	HBD (n)	TPSA	CLogP o/w	Solubility Class	GI Absorption	BBB Permeant	Lipinski Filter	BS
Rule	<500	<10	<10	<5	-	<5	-	-	-	-	-
**C1**	530.89	2	4	0	66.40	3.04	Soluble(p)	High	Yes	Yes(1)	0.55
**C2**	560.92	2	4	2	90.46	2.48	Soluble(p)	High	No	Yes(1)	0.55
**C3**	361.71	1	2	0	36.44	1.87	Soluble(m)	High	Yes	Yes(0)	0.55
**C4**	375.74	2	2	0	36.44	2.14	Soluble(m)	High	Yes	Yes(0)	0.55

mol. wt.—molecular weight; n—number; ROTB—stable bonds; HBA—hydrogen bond acceptors; HBD—hydrogen bond donors; TPSA—topological polar surface area calculated in Å^2^; CLogP o/w—consensus logarithm of partition coefficient between *n*-octanol and water; m—moderate; p—poor; Lipinski filter with number of violations in bracket; GI—gastrointestinal absorption; BBB—blood–brain barrier; BA—bioavailability score.

## Data Availability

The data are contained within the article and Supplementary Materials.

## Supplementary Materials

The following supporting information can be downloaded at https://www.mdpi.com/article/10.3390/ijms25010008/s1. CCDC 2307086 contains the supplementary crystallographic data for this paper. The data are provided free of charge by the Cambridge Crystallographic Data Centre via https://www.ccdc.cam.ac.uk/structures (accessed on 10 November 2023).
