# Supplementary material for: Copper(II) Complexes with 1-(Isoquinolin-3-yl)heteroalkyl-2-ones: Synthesis, Structure and Evaluation of Anticancer, Antimicrobial and Antioxidant Potential
Source: Int J Mol Sci. 2023 Dec 19;25(1):8. doi: 10.3390/ijms25010008 (PMC10779222; doi:10.3390/ijms25010008)
Supplement: Supplementary file 1 [file ijms-25-00008-s001.zip › ijms-2764611-supplementary.pdf]

Supplementary Materials

Copper(II) complexes with 1-(isoquinolin-3-yl)heteroalkyl-2-ones: Synthesis, Structure and Evaluation of Anticancer, Antimicrobial and Antioxidant Potential

Łukasz Balewski [1], Tomasz Plech [2], Izabela Korona-Głowniak [3], Anna Hering [4], Małgorzata Szczesio [5], Andrzej Olczak [5], Patrick J. Bednarski [6], Jakub Kokoszka [1], and Anita Kornicka [1,*]

[1] Department of Chemical Technology of Drugs, Faculty of Pharmacy, Medical University of Gdansk, Gen. J. Hallera 107, 80-416 Gdańsk, Poland; lukasz.balewski@gumed.edu.pl (Ł.B.); anita.kornicka@gumed.edu.pl (A.K); jakub.kokoszka@gumed.edu.pl (J.K.)
[2] Department of Pharmacology, Medical University of Lublin, Radziwiłłowska 11, 20-080 Lublin, Poland; tomasz.plech@umlub.pl (T.P.)
[3] Department of Pharmaceutical Microbiology, Medical University of Lublin, Chodźki 1, 20-093 Lublin, Poland; iza.glowniak@umlub.pl (I.G.)
[4] Department of Biology and Pharmaceutical Botany, Faculty of Pharmacy, Medical University of Gdansk, Gen. J. Hallera 107, 80-416 Gdańsk, Poland; anna.hering@gumed.edu.pl (A.H.)
[5] Institute of General and Ecological Chemistry, Faculty of Chemistry, Lodz University of Technology, Żeromskiego 116, 90-924, Łódź, Poland; malgorzata.szczesio@p.lodz.pl (M.S.)
[6] Department of Pharmaceutical and Medicinal Chemistry, Institute of Pharmacy, University of Greifswald, F.-L. Jahn Strasse 17, D-17489 Greifswald, Germany; bednarsk@uni-greifswald.de (P.J.B.)
* Correspondence: anita.kornicka@gumed.edu.pl (A.K.)

Table of contents:

1. **Infrared spectra**
   Figure S1. IR spectrum of 1-(isoquinolin-3-yl)azetidin-2-one (**L1**) **S3**
   Figure S2. IR spectrum of dichloro{bis[1-(isoquinolin-3-yl)azetidin-2-one]}copper(II) (**C1**) **S3**
   Figure S3. IR spectrum of 1-(isoquinolin-3-yl)imidazolidin-2-one (**L2**) **S4**
   Figure S4. IR spectrum of dichloro{bis[1-(isoquinolin-3-yl)imidazolidin-2-one]}copper(II) (**C2**) **S4**
   Figure S5. IR spectrum of 1-(isoquinolin-3-yl)-3-methylimidazolidin-2-one (**L3**) **S5**
   Figure S6. IR spectrum of dichloro[1-(isoquinolin-3-yl)-3-methylimidazolidin-2-one]copper(II) (**C3**) **S5**
   Figure S7. IR spectrum of 1-ethyl-3-(isoquinolin-3-yl)imidazolidin-2-one (**L4**) **S6**
   Figure S8. IR spectrum of dichloro[1-ethyl-3-(isoquinolin-3-yl)imidazolidin-2-one]copper(II) (**C4**) **S6**
2. **X-ray crystallographic studies**

Table S1. Fractional atomic coordinates and isotropic or equivalent isotropic displacement parameters (Å^2) for dichloro{bis[1-isoquinolin-3-yl)azetidin-2-one]}copper(II) (**C1**) **S7**

Table S2. Atomic displacement parameters (Å^2) for dichloro{bis[1-isoquinolin-3-yl)azetidin-2-one]}copper(II) (**C1**) **S8**

Table S3. Geometric parameters (Å, °) for dichloro{bis[1-isoquinolin-3-yl)azetidin-2-one]}copper(II) (**C1**) **S9**

3. **Stability results**

Figure S9. Time-dependent UV-vis spectra of dichloro{bis[1-(isoquinolin-3-yl)-azetidin-2-one]}copper(II) (**C1**) **S11**

Figure S10. Time-dependent UV-vis spectra of dichloro{bis[1-(isoquinolin-3-yl)-imidazolidin-2-one]}copper(II) (**C2**) **S11**

Figure S11. Time-dependent UV-vis spectra of dichloro[1-(isoquinolin-3-yl)-3-methylimidazolidin-2-one]copper(II) (**C3**) **S12**

Figure S12. Time-dependent UV-vis spectra of dichloro[1-ethyl-3-(isoquinolin-3-yl)imidazolidin-2-one]copper(II) (**C4**) **S12**

4. **Calculated ADME/drug-likeness profiles**

Tables S4. Predicted physicochemical, pharmacokinetic and drug-likeness properties of copper(II) complexes **C1-4** **S13**

Figure S1. IR spectrum of 1-(isoquinolin-3-yl)azetidin-2-one (**L1**)

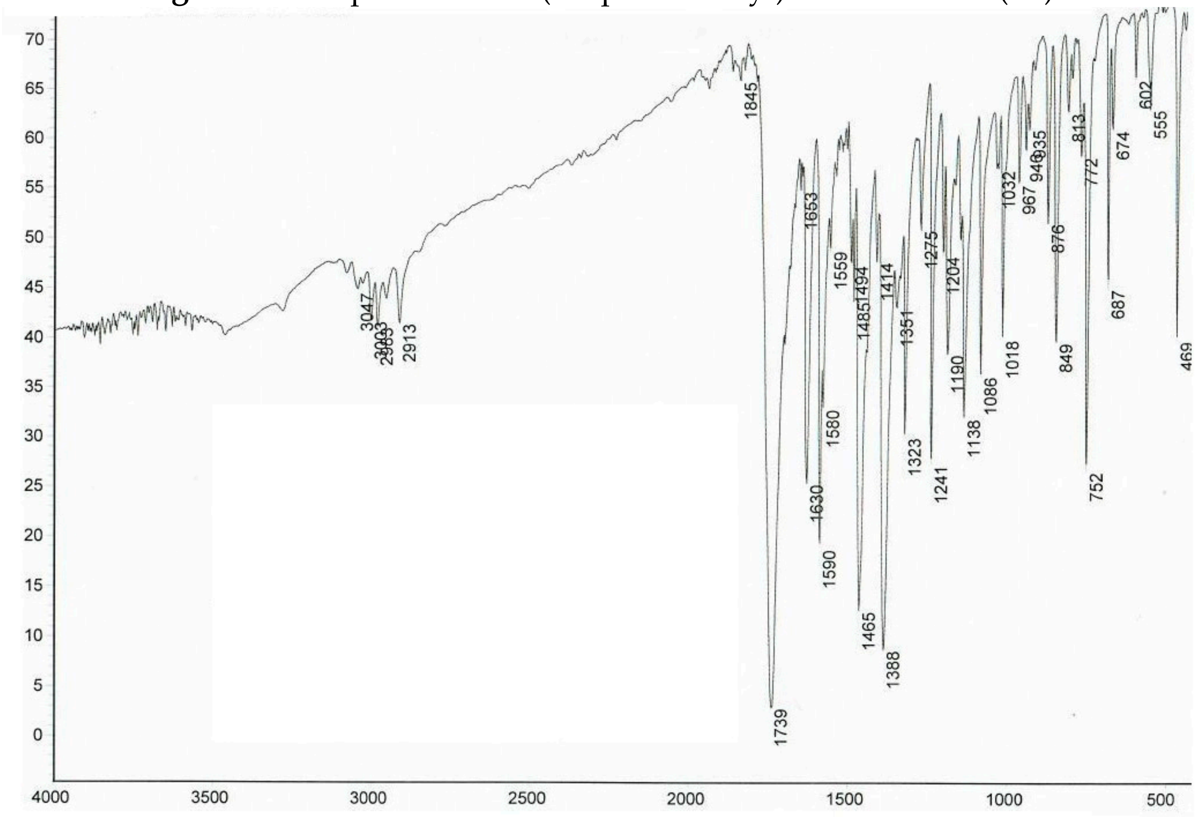

Figure S2. IR spectrum of dichloro{bis[1-(isoquinolin-3-yl)azetidin-2-one]}copper(II) (**C1**)

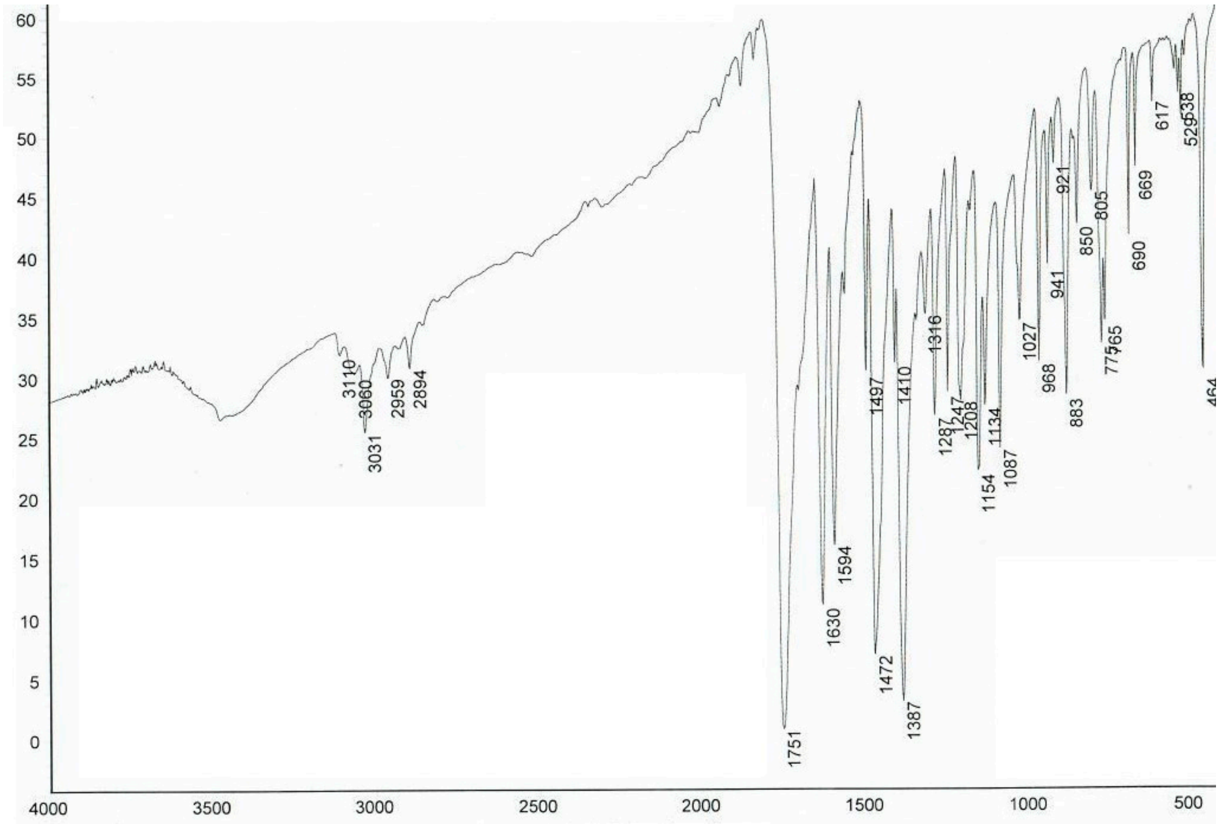

Figure S3. IR spectrum of 1-(isoquinolin-3-yl)imidazolidin-2-one (**L2**)

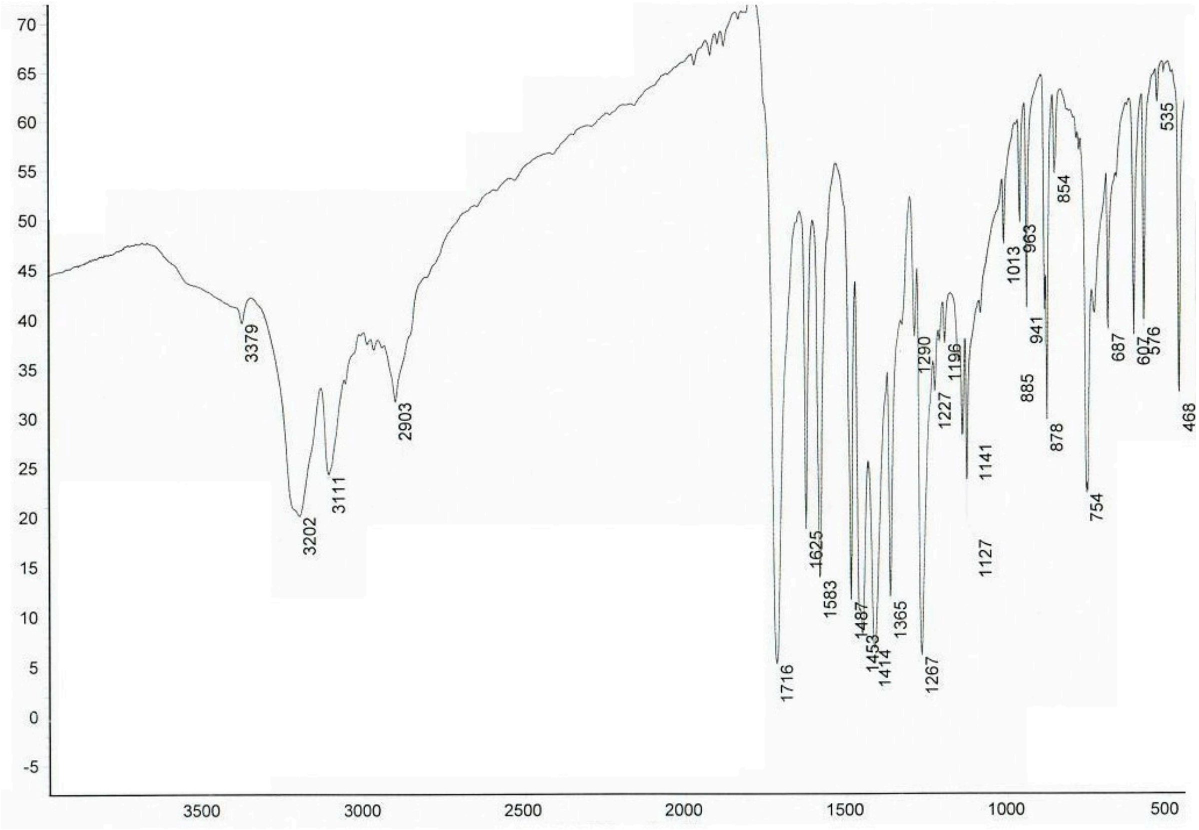

Figure S4. IR spectrum of dichloro{bis[1-(isoquinolin-3-yl)imidazolidin-2-one]}copper(II) (**C2**)

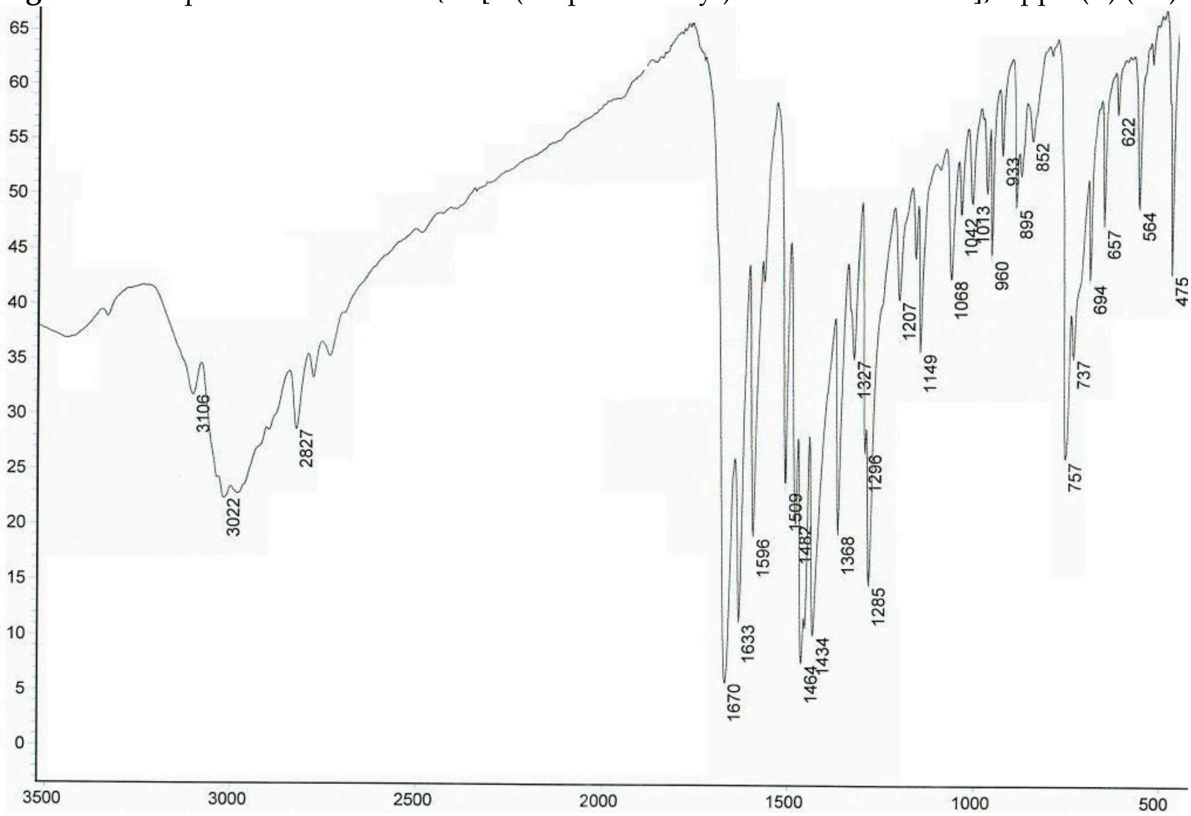

Figure S5. IR spectrum of 1-(isoquinolin-3-yl)-3-methylimidazolidin-2-one (**L3**)

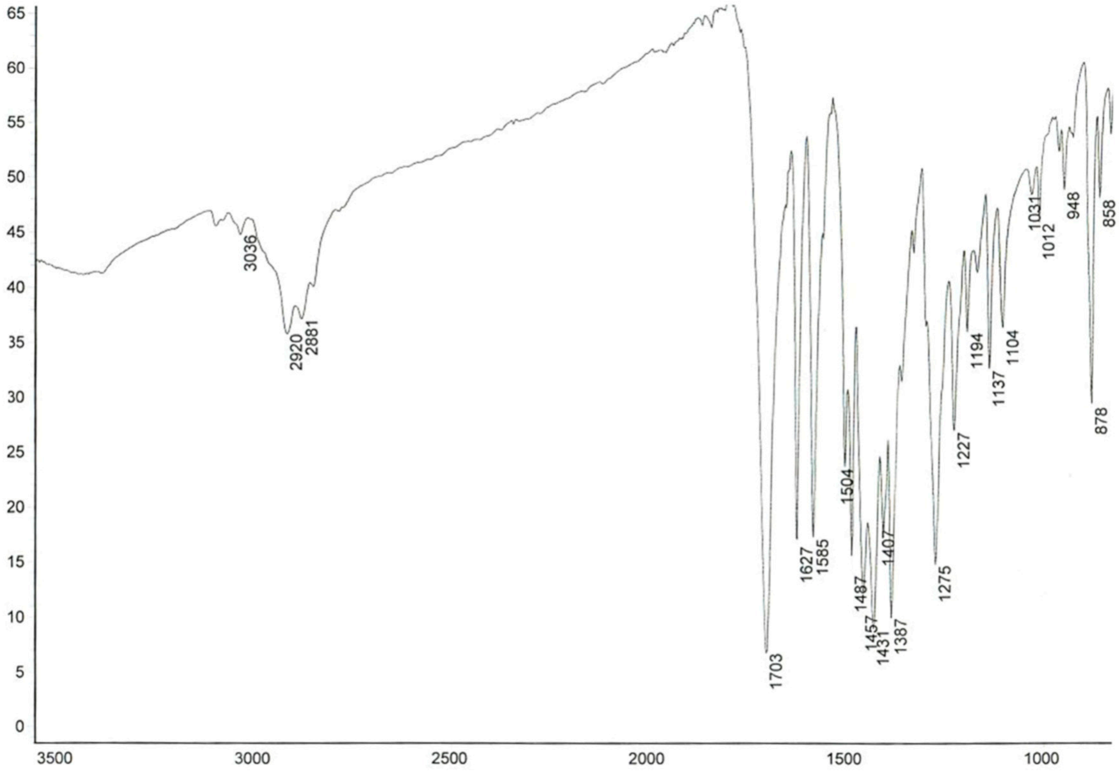

Figure S6. IR spectrum of dichloro[1-(isoquinolin-3-yl)-3-methylimidazolidin-2-one]copper(II) (**C3**)

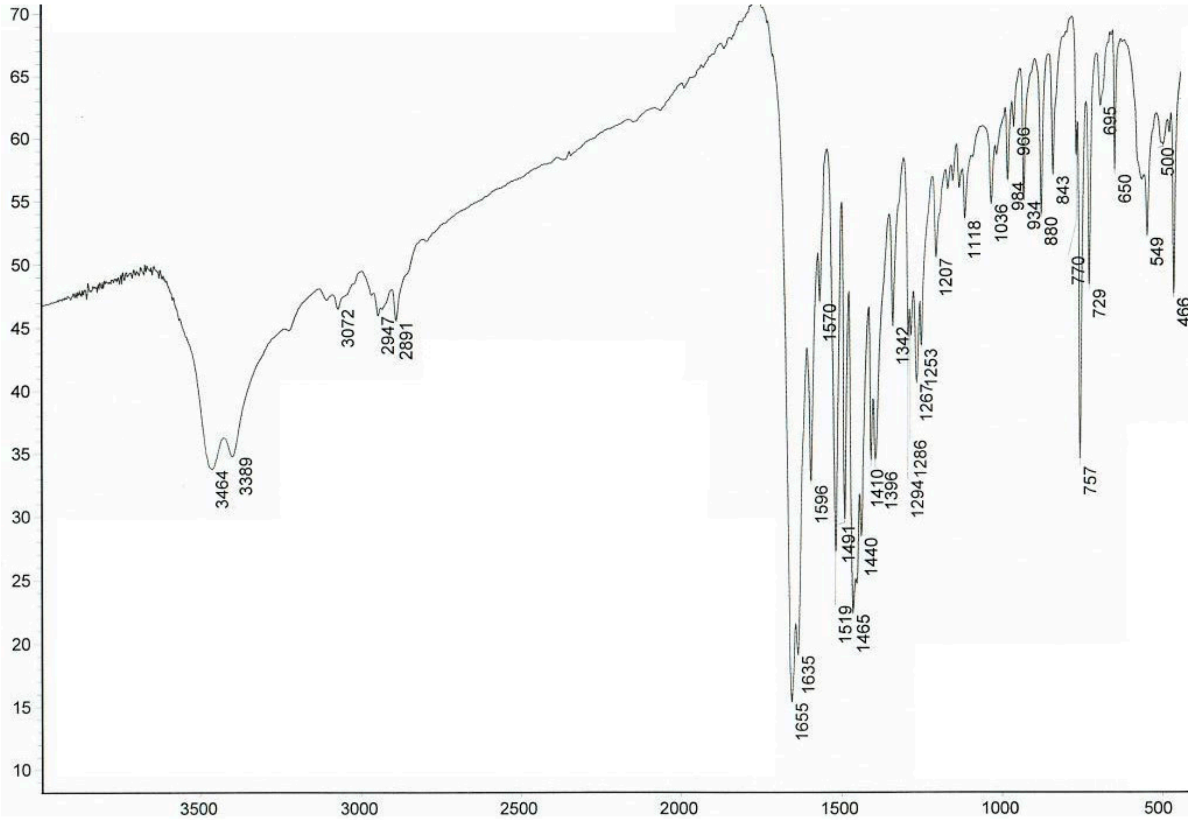

Figure S7. IR spectrum of 1-ethyl-3-(isoquinolin-3-yl)imidazolidin-2-one (**L4**)

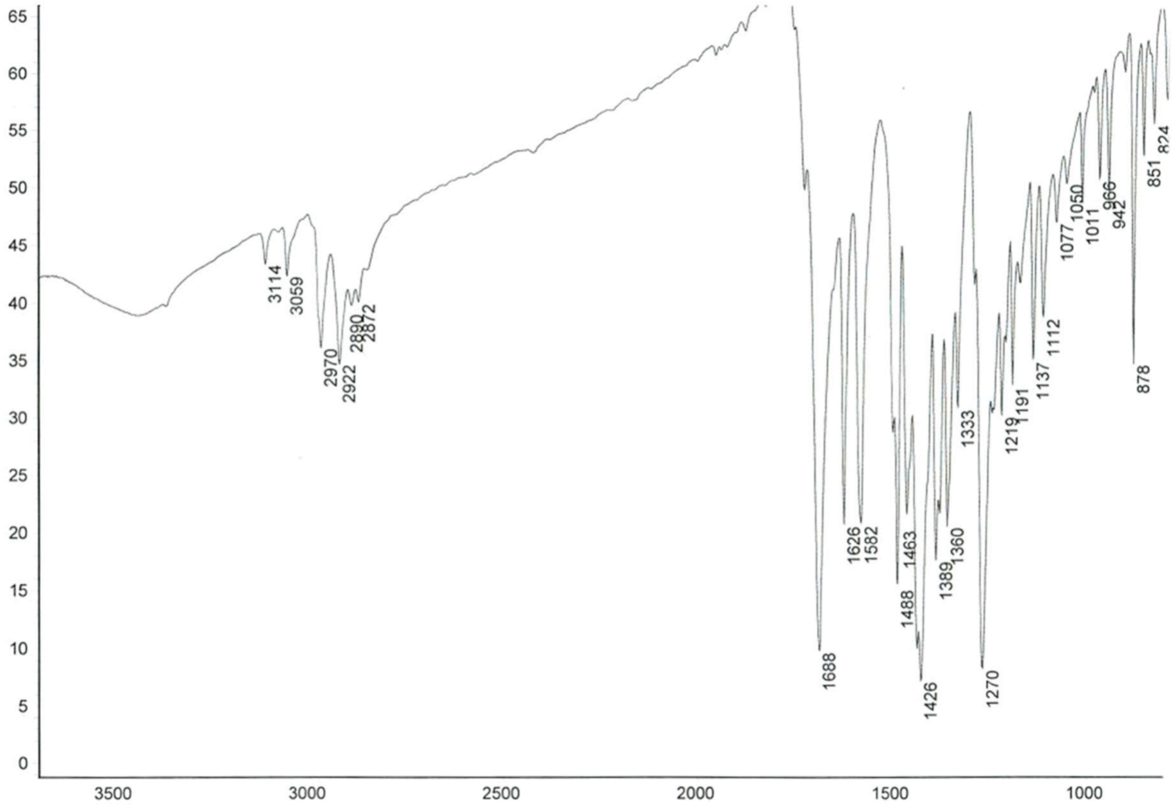

Figure S8. IR spectrum of dichloro[1-ethyl-3-(isoquinolin-3-yl)imidazolidin-2-one]copper(II) (**C4**)

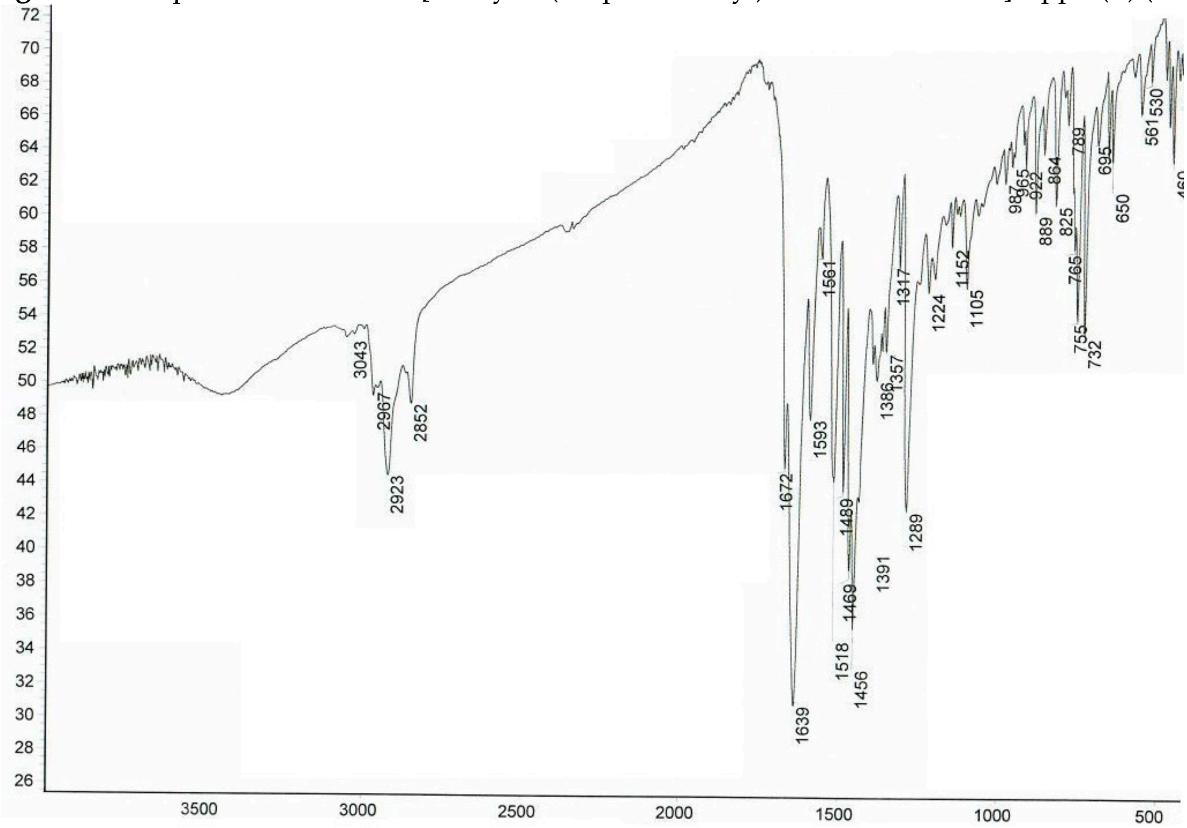

Table S1. Fractional atomic coordinates and isotropic or equivalent isotropic displacement parameters (Å^2) for dichloro{bis[1-isoquinolin-3-yl)azetidin-2-one]}copper(II) (**C1**).

	x	y	z	U_{iso}*/U_{eq}
Cu1	0.500000	0.000000	0.500000	0.00995 (8)
Cl1	0.23838 (5)	-0.12393 (5)	0.52949 (4)	0.01433 (9)
O1	0.28721 (17)	0.02252 (15)	0.25132 (13)	0.0165 (2)
N1	0.50018 (18)	0.24980 (16)	0.62494 (14)	0.0108 (2)
N2	0.24107 (18)	0.27378 (17)	0.41036 (14)	0.0121 (2)
C8	0.5275 (2)	0.58846 (19)	0.82372 (17)	0.0115 (3)
C9	0.3912 (2)	0.51364 (19)	0.66173 (17)	0.0122 (3)
H9	0.307577	0.577491	0.616620	0.015*
C10	0.3803 (2)	0.34690 (19)	0.56935 (16)	0.0105 (3)
C11	0.2070 (2)	0.1368 (2)	0.27445 (17)	0.0123 (3)
C12	0.0401 (2)	0.1894 (2)	0.17116 (17)	0.0141 (3)
H12A	-0.084783	0.103418	0.127707	0.017*
H12B	0.059295	0.225729	0.085859	0.017*
C13	0.0852 (2)	0.3444 (2)	0.32834 (17)	0.0145 (3)
H13A	0.128603	0.461918	0.325388	0.017*
H13B	-0.016877	0.340283	0.366351	0.017*
C2	0.6334 (2)	0.32299 (19)	0.77590 (17)	0.0128 (3)
H2	0.720149	0.258492	0.814485	0.015*
C3	0.6539 (2)	0.49048 (19)	0.88244 (17)	0.0118 (3)
C4	0.7934 (2)	0.5599 (2)	1.04538 (17)	0.0149 (3)
H4	0.880324	0.495567	1.084149	0.018*
C5	0.8021 (2)	0.7199 (2)	1.14637 (18)	0.0170 (3)
H5	0.894685	0.766227	1.255298	0.020*
C6	0.6728 (2)	0.8162 (2)	1.08812 (18)	0.0166 (3)
H6	0.677603	0.925287	1.159760	0.020*
C7	0.5407 (2)	0.7547 (2)	0.93019 (18)	0.0152 (3)
H7	0.458501	0.823030	0.892227	0.018*

Table S2. Atomic displacement parameters (Å^2) for dichloro{bis[1-isoquinolin-3-yl)azetidin-2-one]}copper(II) (**C1**).

	U^{11}	U^{22}	U^{33}	U^{12}	U^{13}	U^{23}
Cu1	0.01242 (13)	0.00748 (12)	0.00888 (12)	0.00298 (9)	0.00399 (9)	0.00188 (9)
Cl1	0.01544 (18)	0.01271 (17)	0.01375 (16)	0.00215 (13)	0.00709 (13)	0.00263 (13)
O1	0.0224 (6)	0.0134 (5)	0.0116 (5)	0.0075 (4)	0.0047 (4)	0.0034 (4)
N1	0.0133 (6)	0.0092 (5)	0.0099 (5)	0.0032 (5)	0.0051 (4)	0.0030 (4)
N2	0.0118 (6)	0.0140 (6)	0.0116 (5)	0.0050 (5)	0.0041 (5)	0.0066 (5)
C8	0.0139 (7)	0.0097 (6)	0.0123 (6)	0.0020 (5)	0.0075 (5)	0.0038 (5)
C9	0.0141 (7)	0.0115 (6)	0.0123 (6)	0.0048 (5)	0.0063 (5)	0.0044 (5)
C10	0.0124 (7)	0.0112 (6)	0.0089 (6)	0.0026 (5)	0.0054 (5)	0.0040 (5)
C11	0.0122 (7)	0.0117 (6)	0.0123 (6)	0.0007 (5)	0.0036 (5)	0.0066 (5)
C12	0.0132 (7)	0.0133 (7)	0.0125 (6)	0.0024 (5)	0.0022 (5)	0.0043 (5)
C13	0.0134 (7)	0.0157 (7)	0.0133 (6)	0.0063 (6)	0.0038 (5)	0.0049 (5)
C2	0.0142 (7)	0.0110 (6)	0.0116 (6)	0.0035 (5)	0.0037 (5)	0.0042 (5)
C3	0.0141 (7)	0.0099 (6)	0.0104 (6)	0.0009 (5)	0.0055 (5)	0.0028 (5)
C4	0.0170 (7)	0.0138 (7)	0.0118 (6)	0.0013 (6)	0.0046 (5)	0.0048 (5)
C5	0.0209 (8)	0.0151 (7)	0.0106 (6)	-0.0027 (6)	0.0066 (6)	0.0018 (5)
C6	0.0225 (8)	0.0111 (7)	0.0149 (7)	-0.0003 (6)	0.0113 (6)	0.0005 (5)
C7	0.0194 (8)	0.0105 (7)	0.0171 (7)	0.0038 (6)	0.0105 (6)	0.0030 (5)

Table S3. Geometric parameters (Å, °) for dichloro{bis[1-isoquinolin-3-yl)azetidin-2-one]}copper(II) (**C1**).

Cu1—Cl1^{i}	2.3051 (4)	C11—C12	1.521 (2)
Cu1—Cl1	2.3051 (4)	C12—H12A	0.9900
Cu1—O1	2.4210 (11)	C12—H12B	0.9900
Cu1—O1^{i}	2.4210 (11)	C12—C13	1.555 (2)
Cu1—N1	2.0666 (12)	C13—H13A	0.9900
Cu1—N1^{i}	2.0666 (12)	C13—H13B	0.9900
O1—C11	1.2139 (19)	C2—H2	0.9500
N1—C10	1.3695 (19)	C2—C3	1.412 (2)
N1—C2	1.3301 (18)	C3—C4	1.423 (2)
N2—C10	1.3971 (18)	C4—H4	0.9500
N2—C11	1.3667 (19)	C4—C5	1.373 (2)
N2—C13	1.4789 (19)	C5—H5	0.9500
C8—C9	1.410 (2)	C5—C6	1.419 (2)
C8—C3	1.416 (2)	C6—H6	0.9500
C8—C7	1.4211 (19)	C6—C7	1.373 (2)
C9—H9	0.9500	C7—H7	0.9500
C9—C10	1.379 (2)		
Cl1^{i}—Cu1—Cl1	180.0	O1—C11—C12	135.88 (14)
Cl1—Cu1—O1	88.91 (3)	N2—C11—C12	92.33 (12)
Cl1^{i}—Cu1—O1	91.09 (3)	C11—C12—H12A	114.3
Cl1—Cu1—O1^{i}	91.09 (3)	C11—C12—H12B	114.3
Cl1^{i}—Cu1—O1^{i}	88.91 (3)	C11—C12—C13	85.96 (11)
O1^{i}—Cu1—O1	180.0	H12A—C12—H12B	111.5
N1—Cu1—Cl1^{i}	88.54 (4)	C13—C12—H12A	114.3
N1^{i}—Cu1—Cl1^{i}	91.46 (4)	C13—C12—H12B	114.3
N1^{i}—Cu1—Cl1	88.54 (4)	N2—C13—C12	86.83 (11)
N1—Cu1—Cl1	91.46 (4)	N2—C13—H13A	114.2
N1—Cu1—O1	89.50 (4)	N2—C13—H13B	114.2
N1^{i}—Cu1—O1	90.50 (4)	C12—C13—H13A	114.2
N1—Cu1—O1^{i}	90.50 (4)	C12—C13—H13B	114.2
N1^{i}—Cu1—O1^{i}	89.50 (4)	H13A—C13—H13B	111.3
N1^{i}—Cu1—N1	180.0	N1—C2—H2	117.9
C11—O1—Cu1	112.59 (9)	N1—C2—C3	124.15 (14)
C10—N1—Cu1	127.35 (10)	C3—C2—H2	117.9
C2—N1—Cu1	115.44 (10)	C8—C3—C4	119.99 (13)
C2—N1—C10	117.21 (12)	C2—C3—C8	117.94 (13)
C10—N2—C13	128.25 (12)	C2—C3—C4	122.05 (14)

C11—N2—C10	136.46 (13)	C3—C4—H4	120.1
C11—N2—C13	94.88 (11)	C5—C4—C3	119.86 (15)
C9—C8—C3	117.84 (13)	C5—C4—H4	120.1
C9—C8—C7	123.09 (14)	C4—C5—H5	120.0
C3—C8—C7	119.05 (13)	C4—C5—C6	120.01 (14)
C8—C9—H9	120.3	C6—C5—H5	120.0
C10—C9—C8	119.41 (14)	C5—C6—H6	119.4
C10—C9—H9	120.3	C7—C6—C5	121.27 (14)
N1—C10—N2	118.23 (12)	C7—C6—H6	119.4
N1—C10—C9	123.37 (13)	C8—C7—H7	120.1
C9—C10—N2	118.39 (13)	C6—C7—C8	119.76 (15)
O1—C11—N2	131.77 (14)	C6—C7—H7	120.1
Cu1—O1—C11—N2	14.7 (2)	C11—N2—C10—N1	-15.7 (2)
Cu1—O1—C11—C12	-167.31 (15)	C11—N2—C10—C9	163.70 (16)
Cu1—N1—C10—N2	-0.52 (19)	C11—N2—C13—C12	0.55 (12)
Cu1—N1—C10—C9	-179.87 (11)	C11—C12—C13—N2	-0.49 (11)
Cu1—N1—C2—C3	-178.19 (11)	C13—N2—C10—N1	173.63 (13)
O1—C11—C12—C13	-177.94 (19)	C13—N2—C10—C9	-7.0 (2)
N1—C2—C3—C8	-1.5 (2)	C13—N2—C11—O1	178.01 (17)
N1—C2—C3—C4	177.08 (15)	C13—N2—C11—C12	-0.56 (12)
N2—C11—C12—C13	0.53 (12)	C2—N1—C10—N2	179.06 (13)
C8—C9—C10—N1	-2.2 (2)	C2—N1—C10—C9	-0.3 (2)
C8—C9—C10—N2	178.47 (13)	C2—C3—C4—C5	-177.01 (14)
C8—C3—C4—C5	1.6 (2)	C3—C8—C9—C10	2.8 (2)
C9—C8—C3—C2	-1.0 (2)	C3—C8—C7—C6	-1.1 (2)
C9—C8—C3—C4	-179.66 (14)	C3—C4—C5—C6	-0.3 (2)
C9—C8—C7—C6	177.61 (15)	C4—C5—C6—C7	-1.7 (2)
C10—N1—C2—C3	2.2 (2)	C5—C6—C7—C8	2.4 (2)
C10—N2—C11—O1	5.3 (3)	C7—C8—C9—C10	-175.99 (14)
C10—N2—C11—C12	-173.24 (17)	C7—C8—C3—C2	177.77 (13)
C10—N2—C13—C12	174.13 (14)	C7—C8—C3—C4	-0.9 (2)

Symmetry code: (i) -*x*+1, -*y*, -*z*+1.

Figure S9. Time-dependent UV-vis spectra
of dichloro{bis[1-(isoquinolin-3-yl)azetidin-2-one]}copper(II) (**C1**)

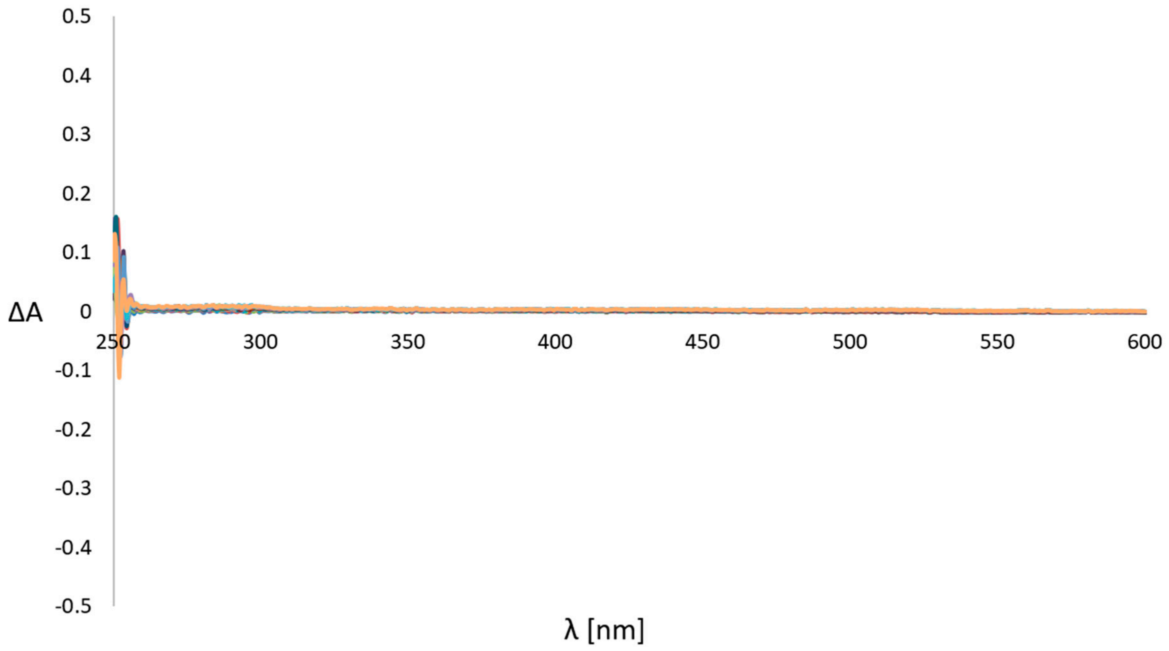

Figure S10. Time-dependent UV-vis spectra
of dichloro{bis[1-(isoquinolin-3-yl)imidazolidin-2-one]}copper(II) (**C2**)

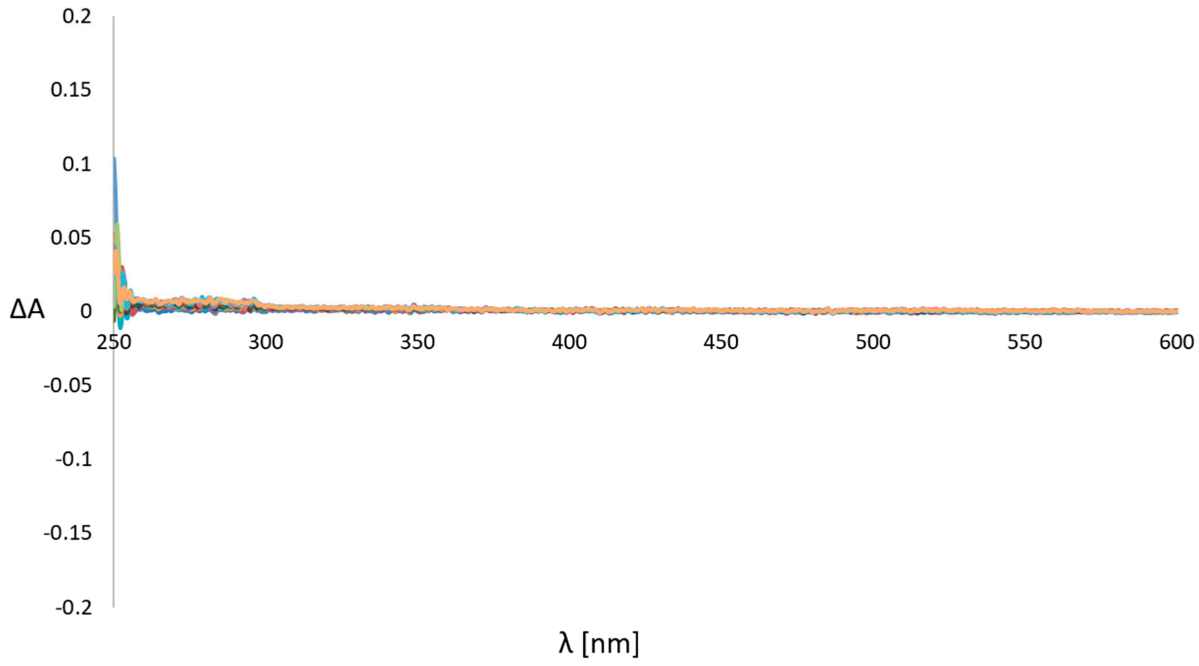

Figure S11. Time-dependent UV-vis spectra of dichloro[1-(isoquinolin-3-yl)-3-methylimidazolidin-2-one]copper(II) (**C3**)

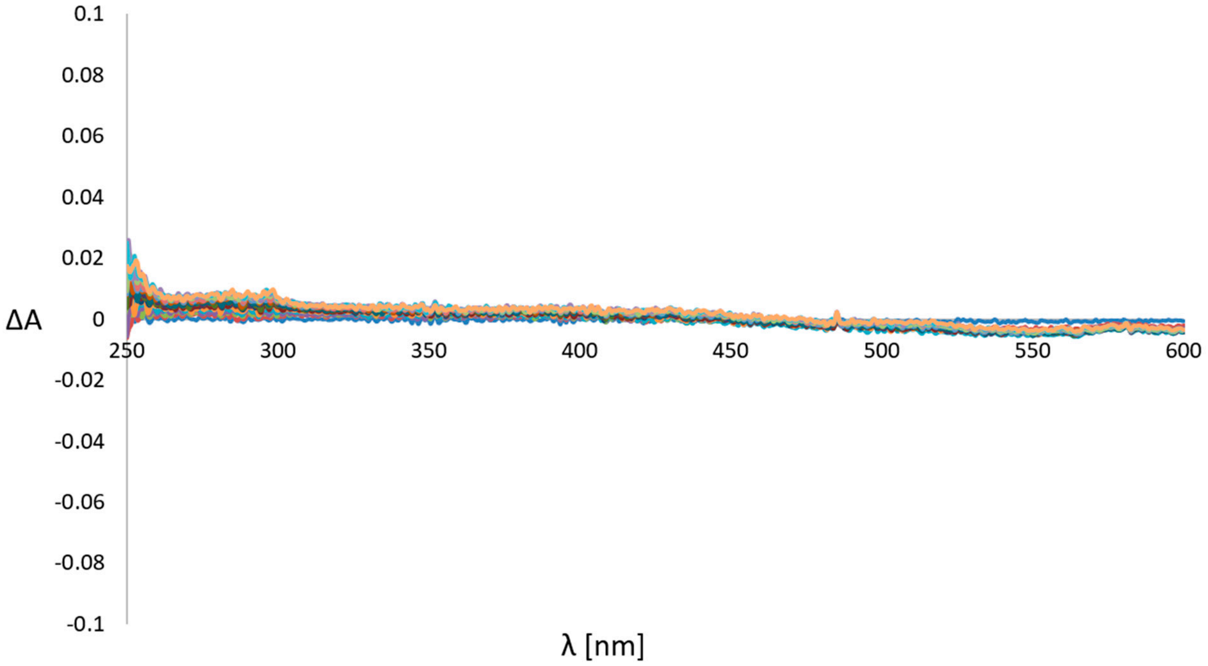

Figure S12. Time-dependent UV-vis spectra of dichloro[1-ethyl-3-(isoquinolin-3-yl)imidazolidin-2-one]copper(II) (**C4**)

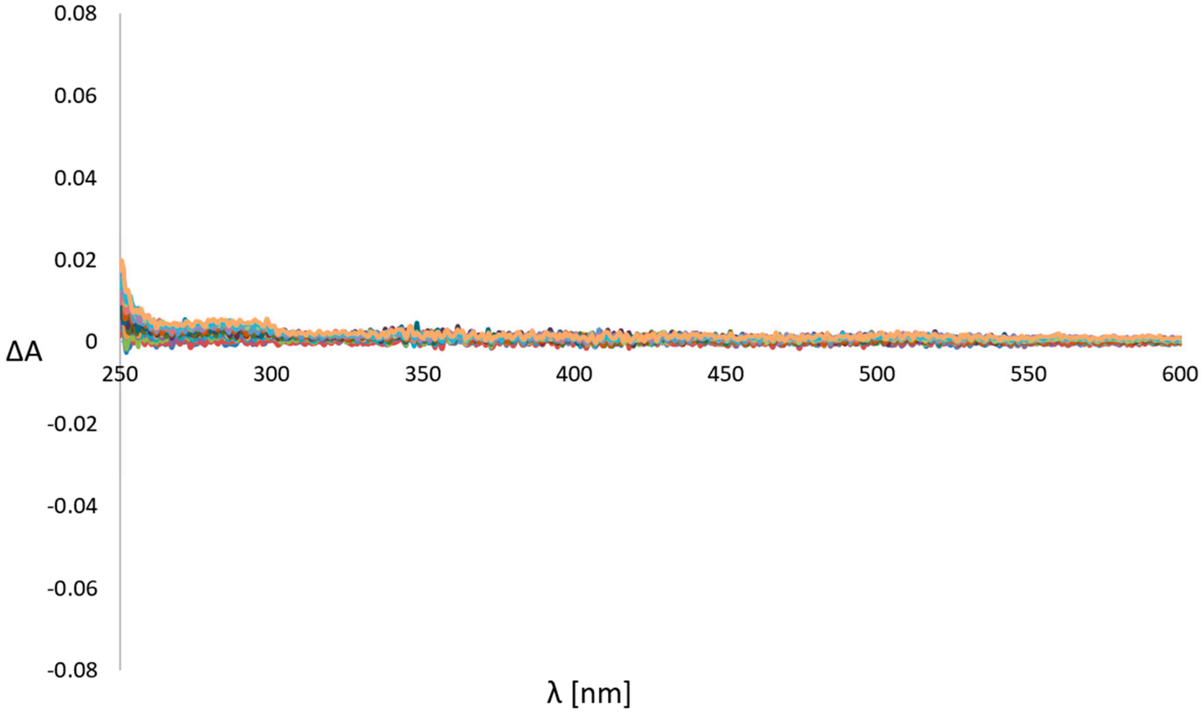

Table S4. Predicted physicochemical, pharmacokinetic and drug-likeness properties of copper(II) complexes **C1-4**

	**Molecule**	**C1**	**C2**	**C3**	**C4**
**Physicochemical Properties**	Formula	$C_{24}H_{20}Cl_2CuN_4O_2$	$C_{24}H_{22}Cl_2CuN_6O_2$	$C_{13}H_{13}Cl_2CuN_3O$	$C_{14}H_{15}Cl_2CuN_3O$
	MW	530.89	560.92	361.71	375.74
	Heavy atoms	33	35	20	21
	Aromatic heavy atoms	20	20	10	10
	Fraction Csp^3	0.17	0.17	0.23	0.29
	Rotatable bonds	2	2	1	2
	H-bond acceptors	4	4	2	2
	H-bond donors	0	2	0	0
	MR	135.26	149.47	85.49	90.30
	TPSA	66.40	90.46	36.44	36.44
**Lipophilicity**	iLOGP	0.00	0.00	0.00	0.00
	XLOGP3	4.61	4.40	3.18	3.55
	WLOGP	4.56	3.38	4.56	3.36
	MLOGP	3.79	3.05	1.99	2.24
	Silicos-IT LogP	2.26	1.55	1.44	1.78
	Consensus LogP	3.04	2.48	1.87	2.14
**Water Solubility**	ESOL Log S	-6.35	-6.38	-4.39	-4.63
	ESOL Solubility (mg/ml)	2.36e-04	2.34e-04	1.47e-02	8.88e-03
	ESOL Solubility (mol/l)	4.44e-07	4.16e-07	4.07e-05	2.36e-05
	ESOL Class	Poorly soluble	Poorly soluble	Moderately soluble	Moderately soluble
	Ali Log S	-5.73	-6.02	-3.62	-4.00
	Ali Solubility (mg/ml)	9.90e-04	5.40e-04	8.75e-02	3.76e-02
	Ali Solubility (mol/l)	1.86e-06	9.62e-07	2.42e-04	9.99e-05
	Ali Class	Moderately soluble	Poorly soluble	Soluble	Moderately soluble
	Silicos-IT LogSw	-3.76	-3.81	-3.49	-3.89
	Silicos-IT Solubility (mg/ml)	9.23e-02	8.61e-02	1.18e-01	4.83e-02

	Silicos-IT Solubility (mol/l)	1.74e-04	1.53e-04	3.26e-04	1.29e-04
	Silicos-IT class	Soluble	Soluble	Soluble	Soluble
**Pharmacokinetics**	GI absorption	High	High	High	High
	BBB permeant	Yes	No	Yes	Yes
	Pgp substrate	Yes	Yes	Yes	Yes
	CYP1A2 inhibitor	No	No	No	No
	CYP2C19 inhibitor	No	Yes	Yes	Yes
	CYP2C9 inhibitor	No	No	No	No
	CYP2D6 inhibitor	No	No	No	No
	CYP3A4 inhibitor	No	No	No	No
	log Kp (cm/s)	-6.27	-6.60	-6.25	-6.07
**Drug-likeness**	Lipinski violations	1	1	0	0
	Ghose violations	2	2	0	0
	Veber violations	0	0	0	0
	Egan violations	0	0	0	0
	Muegge violations	0	0	0	0
	Bioavailability Score	0.55	0.55	0.55	0.55
**Medicinal Chemistry**	PAINS alerts	0	0	0	0
	Brenk alerts	0	0	0	0
	Leadlikeness violations	2	2	1	2
	Synthetic Accessibility	2.73	3.33	2.46	2.56
